# Boosting Chemiexcitation of Phenoxy-1,2-dioxetanes through 7-Norbornyl and Homocubanyl Spirofusion

**DOI:** 10.1021/jacsau.4c00493

**Published:** 2024-09-03

**Authors:** Sara Gutkin, Omri Shelef, Zuzana Babjaková, Laura Anna Tomanová, Matej Babjak, Tal Kopp, Qingyang Zhou, Pengchen Ma, Micha Fridman, Urs Spitz, Kendall N. Houk, Doron Shabat

**Affiliations:** † School of Chemistry, Raymond and Beverly Sackler Faculty of Exact Sciences, Tel-Aviv University, Tel Aviv 69978, Israel; ‡ Biosynth, Rietlistr. 4 Postfach, Staad 125 9422, Switzerland; § Department of Organic Chemistry, Slovak University of Technology in Bratislava, Radlinskeho 9, Bratislava 81237, Slovakia; ∥ Department of Chemistry and Biochemistry, University of California, Los Angeles, California 90095, United States; ⊥ Department of Chemistry, School of Chemistry, Xi’an Key Laboratory of Sustainable Energy Material Chemistry and Engineering Research Center of Energy Storage Materials and Devices, Ministry of Education, Xi’an Jiaotong University, Xi’an 710049, China

**Keywords:** chemiluminescence, 1,2-dioxetanes, spiro-fused compounds, enzyme detection, molecular probes

## Abstract

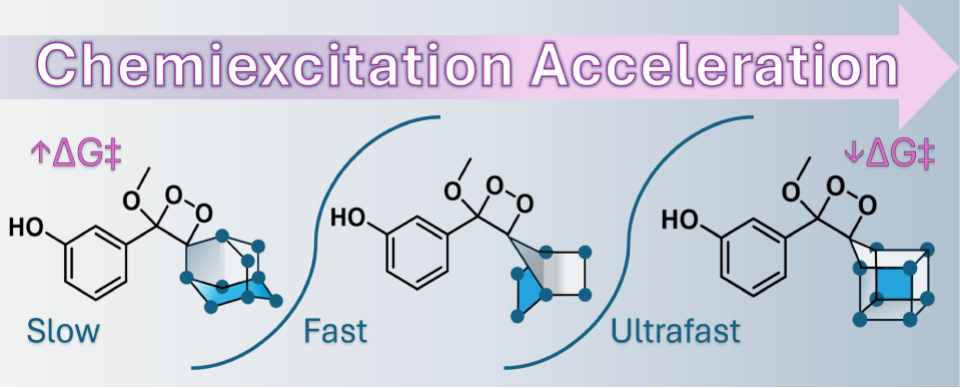

The chemiluminescent light-emission pathway of phenoxy-1,2-dioxetane luminophores is increasingly attracting the scientific community’s attention. Dioxetane probes that undergo rapid, flash-type chemiexcitation demonstrate higher detection sensitivity than those with a slower, glow-type chemiexcitation rate. This is primarily because the rapid flash-type produces a greater number of photons within a given time. Herein, we discovered that dioxetanes fused to 7-norbornyl and homocubanyl units present accelerated chemiexcitation rates supported by DFT computational simulations. Specifically, the 7-norbornyl and homocubanyl spirofused dioxetanes exhibited a chemiexcitation rate 14.2-fold and 230-fold faster than that of spiro-adamantyl dioxetane, respectively. A turn-ON dioxetane probe for the detection of the enzyme β-galactosidase, containing the 7-norbornyl spirofused unit, exhibited an S/N value of 415 at a low enzyme concentration. This probe demonstrated an increase in detection sensitivity toward β-galactosidase expressing bacteria *E. coli* with a limit-of-detection value that is 12.8-fold more sensitive than that obtained by the adamantyl counterpart. Interestingly, the computed activation free energies of the homocubanyl and 7-norbornyl units were correlated with their CC_s_C spiro-angle to corroborate the measured chemiexcitation rates.

Controlling the chemiexcitation rate of 1,2-dioxetanes enables to manufacture chemiluminophores capable of emitting light in either a continuous glow or a rapid flash mode.^1,2^ Flash-type chemiluminescence assays outperform glow-type assays by generating more intense light emission signals, primarily due to the higher photon count produced within a specified time interval.^3−8^ The chemiexcitation pathway of Schaap’s phenoxy 1,2-dioxetanes is initiated through an electron transfer from a phenolate species to a peroxide bond of a spiro-cycloalkyl-dioxetane.^9,10^ This event leads to a disassembly of the dioxetane and the generation of an excited benzoate species that decays to its ground state through the emission of a photon. Since 1,2-dioxetanes are chemically unstable, a spiro-adamantyl unit is incorporated in the molecule, in order to elevate its stability through the generation of a steric hindrance.^11−22^

We have recently shown that the chemiexcitation of phenoxy-1,2-dioxetanes can be significantly accelerated through a spiro-strain release effect resulting in replacing the traditional adamantyl unit with a cyclobutyl molecular motif.^23^ The acceleration of 1,2-dioxetane chemiexcitation enabled the production of chemiluminescent probes with an extremely high signal-to-noise, which exhibited unprecedented detection sensitivity. The discovery of the spiro-strain release effect stimulated us to evaluate the influence of other bridged-cycloalkyl units on the chemiexcitation rate of phenoxy 1,2-dioxetanes.^24^ Here, we report a chemiexcitation acceleration effect for phenoxy 1,2-dioxetanes generated by a spiro-fused effect of polycyclic homocubanyl and bicyclic 7-norbornyl units (Figure 1).

**Figure 1:**
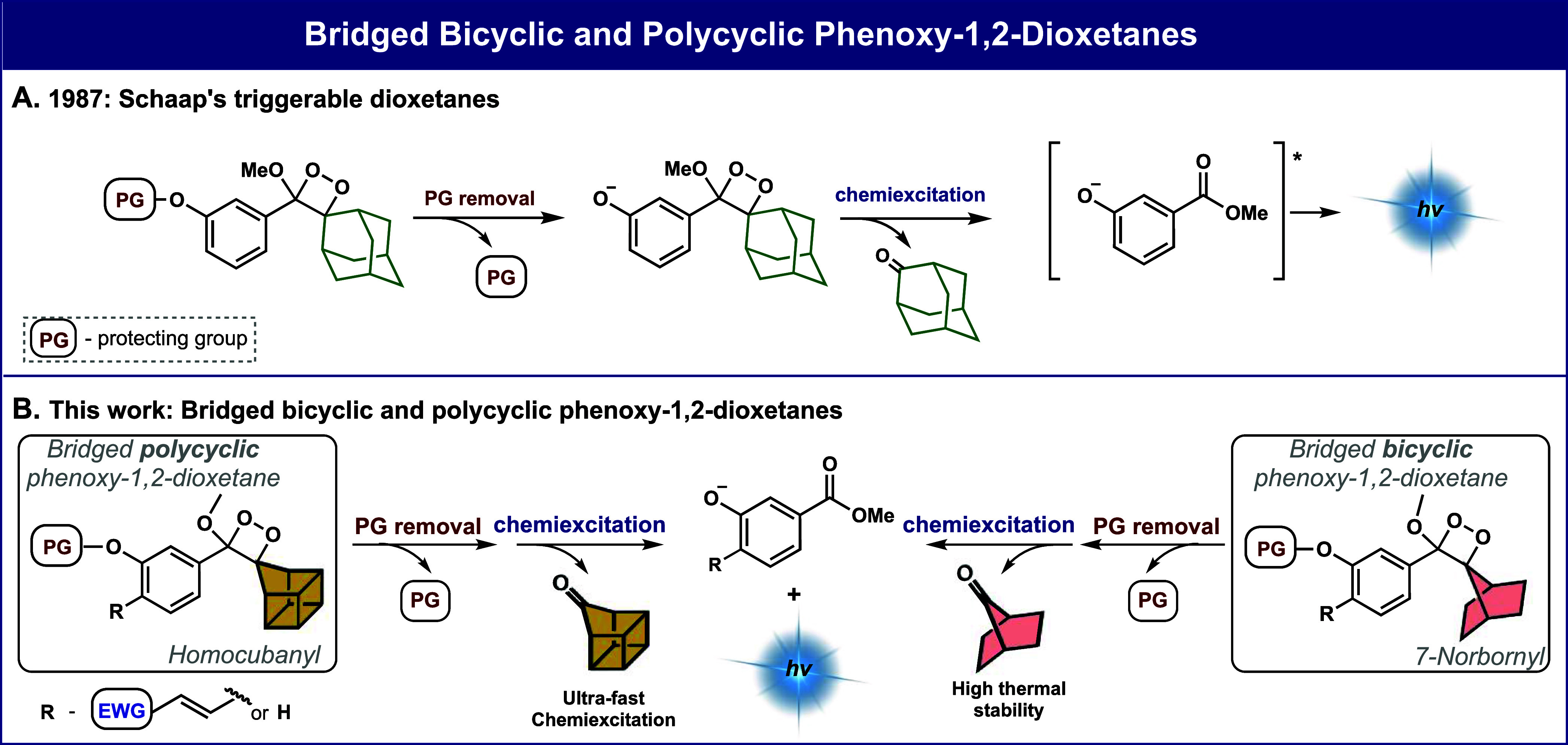
A) Activation and chemiexcitation pathway of adamantyl-phenoxy-1,2-dioxetanes. B) Chemiexcitation pathway of a general bridged cyclic homocubanyl and 7-norbornyl phenoxy-dioxetanes presented in this work.

The rate-determining step of phenoxy-1,2-dioxetane chemiexcitation is the O–O cleavage of the dioxetane that is accompanied by electron transfer from the phenolate to the dioxetane to generate a biradical species.^13^ We hypothesized that by exchanging the spiro-adamantyl-dioxetane unit with a bridged bicyclic or polycyclic unit with a constrained angle, the additional strain at the spirofusion could lead to faster O–O cleavage and accelerated chemiexcitation.^25−27^ We explored this hypothesis and performed quantum mechanical studies with reliable DFT methods (see the Supporting Information for details). The computational results in Figure 2 indicate that the adamantyl-phenoxy-1,2-dioxetane exhibits a comparatively slow chemiexcitation rate, predicted by the relatively high barrier of 18.2 kcal/mol (Figure 2B) for the rate-determining transition state with O–O cleavage and partial electron transfer. By contrast, the 7-norbornyl and homocubanyl-phenoxy-1,2-dioxetanes have faster chemiexcitation rates due to barriers of only 16.9 and 14.2 kcal/mol, respectively (Figure 2B). The computational results of the rate-determining step (O–O cleavage transition state) for four phenoxy-1,2-dioxetanes are compared in Figure 2B.

**Figure 2:**
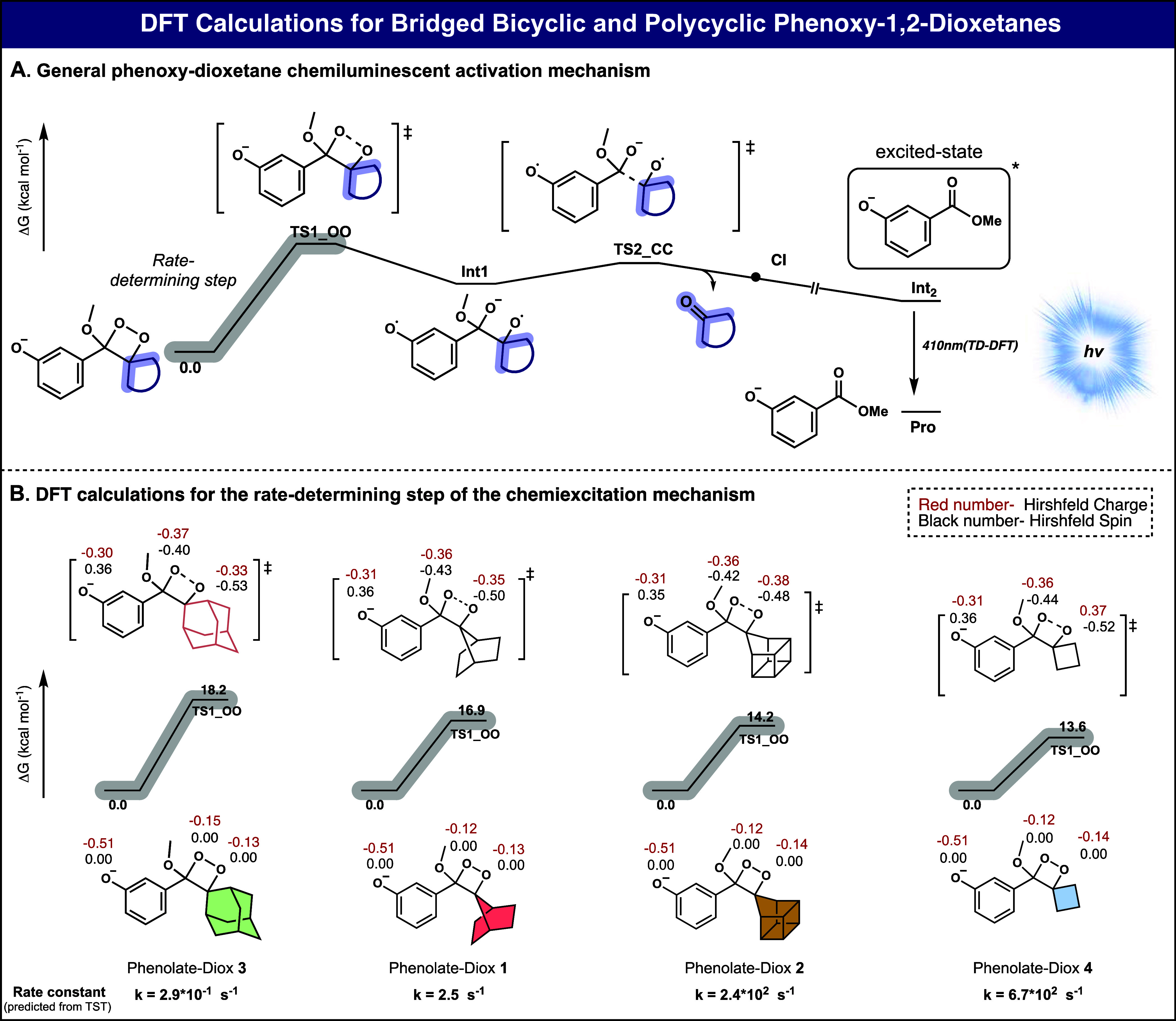
A) The chemiexcitation mechanism of a general spiro-cycloalkyl phenoxy-1,2-dioxetane. B) Comparison between the computed Gibbs free energy of the rate-determining step of chemiexcitation for four selected phenoxy-1,2-dioxetane. Detailed DFT calculations are presented in the (Figures S1 and S2).

The bicyclic 7-norbornyl-phenoxy 1,2-dioxetane, Diox 1, and the polycyclic-homocubanyl phenoxy 1,2-dioxetane, Diox 2 (Table 1), were synthesized in a similar manner reported for other phenoxy 1,2-dioxetanes (see Supporting Information). Adamantyl and cyclobutyl phenoxy-1,2-dioxetanes, Diox 3 and Diox 4 were used as reference control compounds. To facilitate the measuring of Diox 1–Diox 4 chemiluminescent properties, the phenol functional groups of the dioxetanes were masked with a *tert*-butyl-dimethyl silyl (TBS) triggering group. The chemiexcitation of the dioxetanes was initiated by removing the TBS groups via tetra-butyl-ammonium-fluoride (TBAF) addition. The molecular structures of the four dioxetanes, their stability under physiological conditions (PBS 7.4, RT), total light emission half-lives (*t*_1/2_) in DMSO or acetone, and relative chemiexcitation rate, are presented in Table 1. Previous measurements by our group have shown that the chemiexcitation rate of 1,2-dioxetanes is particularly fast in polar organic solvents like DMSO, but the rate can be modulated by selecting alternative solvents.^23^ Therefore, measurements of *t*_1/2_ values for the total light emission of dioxetanes exhibiting relatively slow chemiexcitation rates were determined in DMSO, while measurements for dioxetanes with faster chemiexcitation rates were conducted in acetone.^28^

**Table 1:**
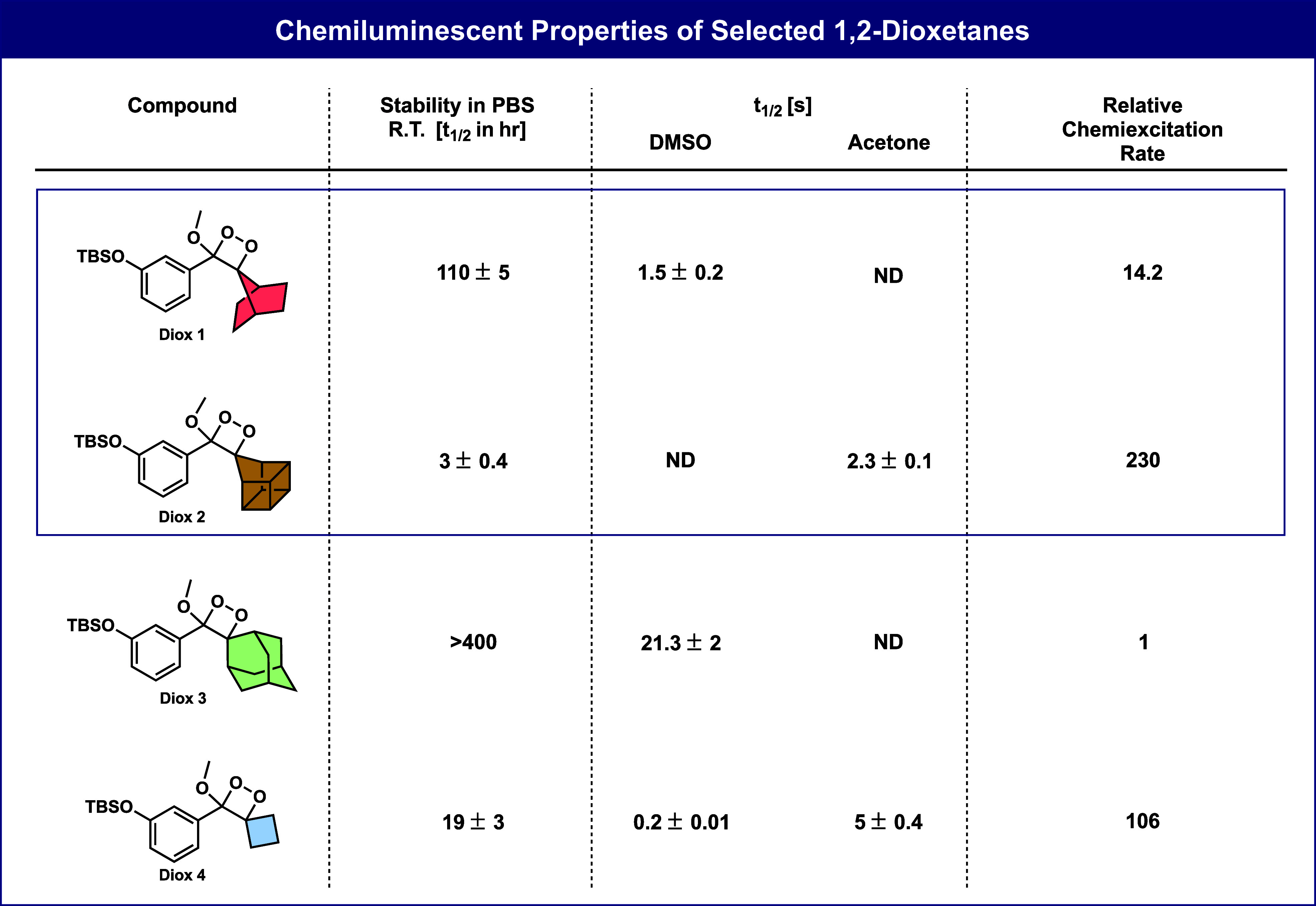
Molecular Structures and Chemiluminescent Properties of Diox 1–Diox 4^a^

a The stability of Diox 1–Diox 4 [500 μM] was measured in PBS, pH 7.4, 15% ACN at 25 °C; product distribution was determined using RP-HPLC (90–100% ACN in water with 0.1% TFA). Chemiexcitation properties of Diox 1–Diox 4 [10 nM] were measured in DMSO or acetone, with TBAF [10 mM], with 10% ACN. Measurements were performed in triplicate using independent samples. All measurements were conducted using SpectraMax iD3, with injector settings fixed on an integration time of 50 ms. The *t*_1/2_ of Diox 3 in DMSO was used as a reference. Relative chemiexcitation rate is defined as the ratio between the *t*_1/2_ values of Diox 1–Diox 4. Half-life value (*t*_1/2_) is defined as the time point by which half of the total light emission was observed.

The relative chemiexcitation rates of the four dioxetanes, Diox 1, Diox 2, Diox 3, and Diox 4, were determined by evaluating their total light emission *t*_1/2_ values according to the plots presented in the (Figures S3–S5). Both Diox 1 and Diox 2 exhibited a notably enhanced chemiexcitation rate compared to the spiro-adamantyl dioxetane, Diox 3 (14.2-fold and 230-fold, respectively). Intriguingly, the chemiexcitation rate of the homocubanyl dioxetane surpassed even that of the cyclobutyl-dioxetane, Diox 4, by 2-fold.

A visual demonstration of the chemiexcitation acceleration effect obtained by the bridged bicyclic and polycyclic units in Diox 1 and Diox 2, is presented in Figure 3A. Images taken at selected time intervals over 90 s show the light emission of Diox 1–Diox 4 in acetone as a solvent. The 7-norbornyl-dioxetane Diox 1 emitted light with a chemiexcitation rate that is significantly faster than that of its adamantyl analog Diox 3, lasting beyond 15 s but less than 90 s. The homocubanyl dioxetane, Diox 2, displayed an ultrafast chemiexcitation rate, lasting for less than 15 s (*t*_1/2_ = 3 s). This chemiexcitation rate is about 2-fold faster compared to that of the cyclobutyl-dioxetane, Diox 4 (*t*_1/2_ = 5 s). Normalized plots of the four dioxetanes showing their relative chemiexcitation rates are presented in Figure 3B.

**Figure 3:**
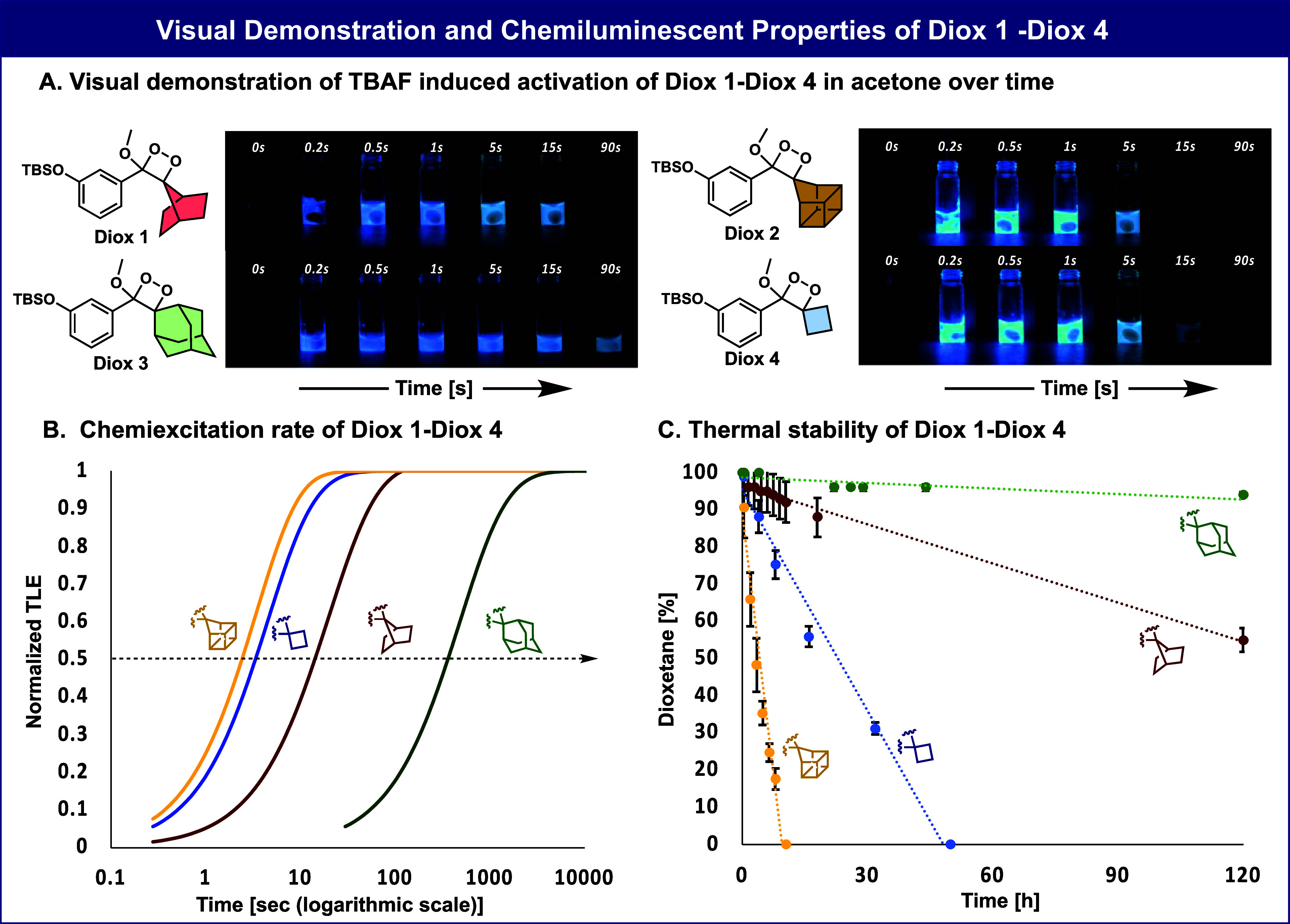
A) Molecular structures and visual demonstration of the light emitted by Diox 1–Diox 4 [500 μM] during 90 s in the presence of TBAF [10 mM] in acetone. B) Normalized total light emission kinetic profile (time is represented in logarithmic scale) of Diox 1–Diox 4. The relative calculated chemiexcitation rates are taken from Table 1 and Figures S3–S5. C) Chemical stability of Diox 1–Diox 4 [500 μM] was measured in PBS [100 mM], pH 7.4, 10% ACN at 25 °C; decomposition products were determined using RP-HPLC (90–100% ACN in water with 0.1% TFA). See chemical stability values of Diox 1–Diox 4 in Figure S9. Measurements were performed in triplicate using independent samples.

The chemical stabilities of the four dioxetanes were determined by monitoring the spontaneous decomposition over time at room temperature, in PBS, pH 7.4 (Figure 3C). Diox 1 and Diox 2 were found to be less stable than their parent adamantyl derivative. This phenomenon is attributed to the increased strain existing in the spiro bicyclic and polycyclic dioxetane units.^26,29−31^ However, the 7-norbornyl dioxetane, Diox 1, exhibited substantially higher stability than the cyclobutyl derivative Diox 4. The homocubanyl derivative Diox 2, exhibited very low stability (*t*_1/2_ = 3h) rendering it unsuitable for further application in a biological context.

The rapid chemiexcitation observed for phenoxy-1,2-dioxetanes containing bridged cyclic units suggests that a turn-ON probe utilizing such a luminophore is anticipated to exhibit higher detection sensitivity. While the homocubanyl moiety exhibited a significantly faster chemiexcitation rate than the 7-norbornyl counterpart, its instability hinders its application in pseudobiological and biological assays. We have previously shown that the incorporation of an acrylate substituent at the *ortho*-position of phenoxy-1,2-dioxetane generates a chemiluminophore, which is extremely emissive under physiological conditions.^32^ This chemiluminophore was demonstrated to be highly useful for constructing turn-on probes for the detection and imaging of various enzymes and bioanalytes.^5−7,33−37^ Therefore, we next synthesized a new *ortho*-acrylate substituted phenoxy-1,2-dioxetane chemiluminescent probe, with a 7-norbornyl motif (probe MA-β-gal-norbornyl), for the detection of β-galactosidase (β-gal) enzymatic activity (Figure 4A).^38^ The probe’s activity was compared with that of the known adamantyl-1,2-dioxetane (probe MA-β-gal-adamantyl).

**Figure 4:**
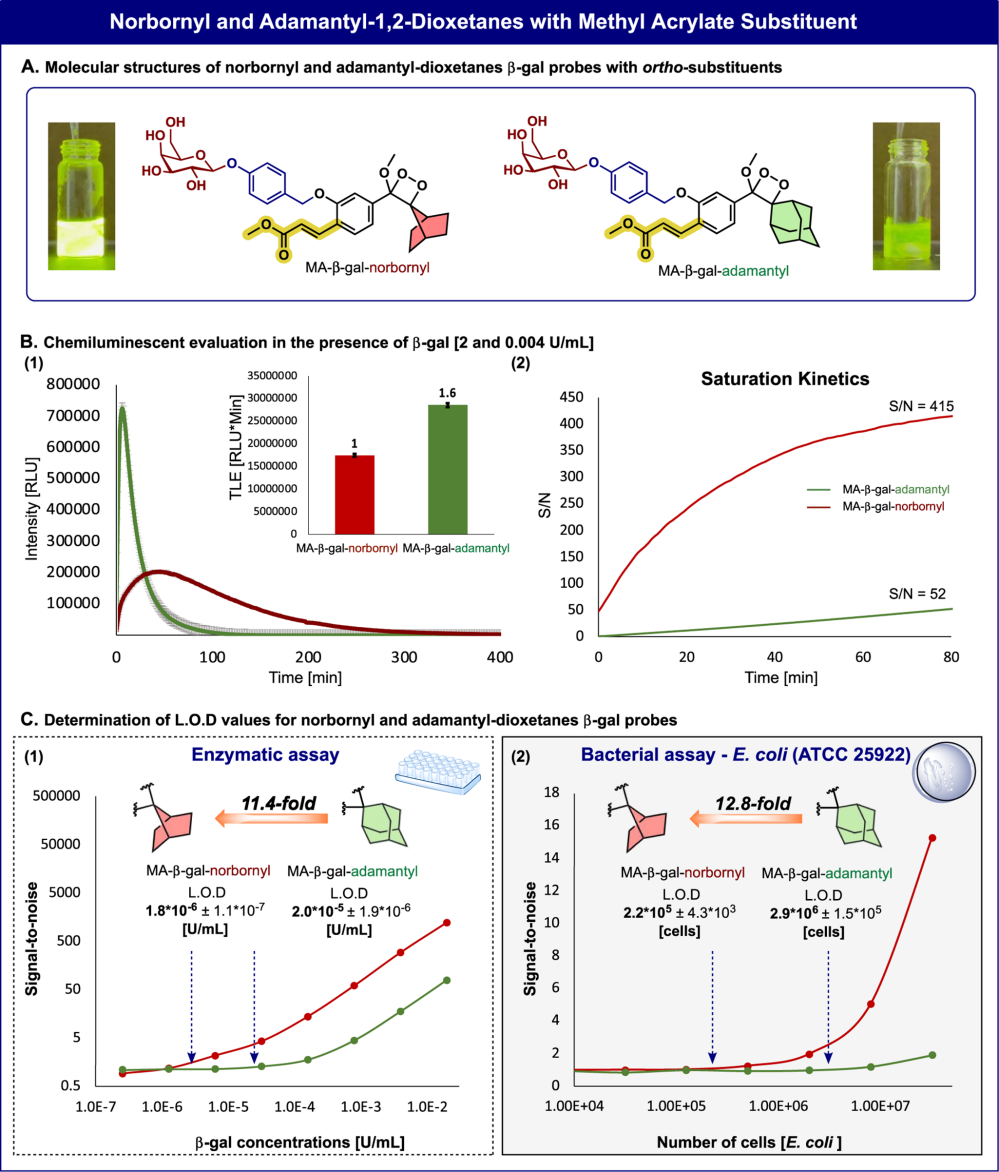
A) Molecular structures of 7-norbornyl and adamantyl methyl acrylate probes. B) (1) Chemiluminescence kinetic profiles and total light emission (inset) of MA-β-gal-norbornyl and MA-β-gal-adamantyl [10 μM] in the presence and absence of β-gal [2 U/mL]. (2) Chemiluminescence signal-to-noise over time of MA-β-gal-norbornyl and MA-β-gal-adamantyl [10 μM] in the presence and absence of β-gal [0.004 U/mL]. C) (1) Determination of the limit of detection values: Signal-to-noise of total light emission of MA-β-gal-norbornyl and MA-β-gal-adamantyl [10 μM] in the presence and absence of different β-gal concentrations [2.56 × 10^–7^–1 × 10^–3^ U/mL] (presented in a logarithmic scale) after 30 min of measurements. All measurements were conducted in PBS, pH 7.4, with 10% ACN at 27 °C. (Figures S10–S14). (2) Determination of the limit of detection values: signal-to-noise of total light emission of MA-β-gal-norbornyl and MA-β-gal-adamantyl [10 μM] in the presence and absence of different concentrations of *E. coli* ATCC 25922 [7.98 × 10^3^ to 3.20 × 10^7^ cells]. All measurements were conducted in PBS, pH 7.4, with 0.1% ACN at 37 °C. (Figures S15–S16). Measurements were performed in triplicate using independent samples.

The full light emission profiles of probe MA-β-gal-norbornyl and probe MA-β-gal-adamantyl, in the presence of a high concentration of β-gal [2 U/mL] in PBS 7.4, are presented in Figure 4B1. The relative chemiluminescence quantum yields of the probes were determined by measuring the total light emission generated upon activation with β-gal (Figure 4B1, inset). Predictably, probe MA-β-gal-norbornyl exhibited a rapid and intense light emission response that decayed after 100 min. On the other hand, the light emission profile of probe MA-β-gal-adamantyl was less intense and lasted for over 300 min.

Next, the light emission signals of probes MA-β-gal-norbornyl and MA-β-gal-adamantyl were evaluated under low enzyme concentrations. Under such conditions, the signal is gradually increased to reach a plateau level, which lasts for a long period. The signal-to-noise (S/N) of the plateau signal generated by probe MA-β-gal-norbornyl, was substantially higher than the S/N value produced by probe MA-β-gal-adamantyl, after 80 min; 415 and 52 respectively (Figure 4B2). The detection sensitivity of the two probes toward β-gal was determined by measuring the light emission signal over a varied range of enzyme concentrations (Figure 4C1) and with various optical densities of β-gal-expressing bacterial strain *Escherichia coli* ATCC 25922 (Figure 4C2). Predictably, the limit-of-detection value (LOD) obtained for the enzymatic assay by probe MA-β-gal-norbornyl (1.8 × 10^–6^ U/mL) was 11.4-fold lower than the LOD value achieved by probe MA-β-gal-adamantyl (2.0 × 10^–5^ U/mL). Similarly, the LOD value obtained by probe MA-β-gal-norbornyl (2.2 × 10^5^ cells) in bacterial cell assay was 12.8-fold lower than the LOD value achieved by probe MA-β-gal-adamantyl (2.9 × 10^6^ cells). This data indicates that probe MA-β-gal-norbornyl has a 12.8-fold higher detection sensitivity for β-gal activity in bacteria.

We next performed additional DFT calculations to understand if the strain of the spirocycles, measured as the CC_s_C angle (C_s_ is the spiro-carbon) relates to the rate of chemiexcitation. Figure 5A shows that the computed activation free energies correlate reasonably well with this angle, with the homocubanyl reacting faster than expected based on the spiro angle alone. We have also included the rate calculated for the dimethyl-substituted dioxetane, which has no spirostrain. Figure 5B plots the log of the measured relative emission rates versus these same spiro angles. Once again, homocubanyl is an outlier, reacting faster than expected, based on the spiro fusion angles.

**Figure 5:**
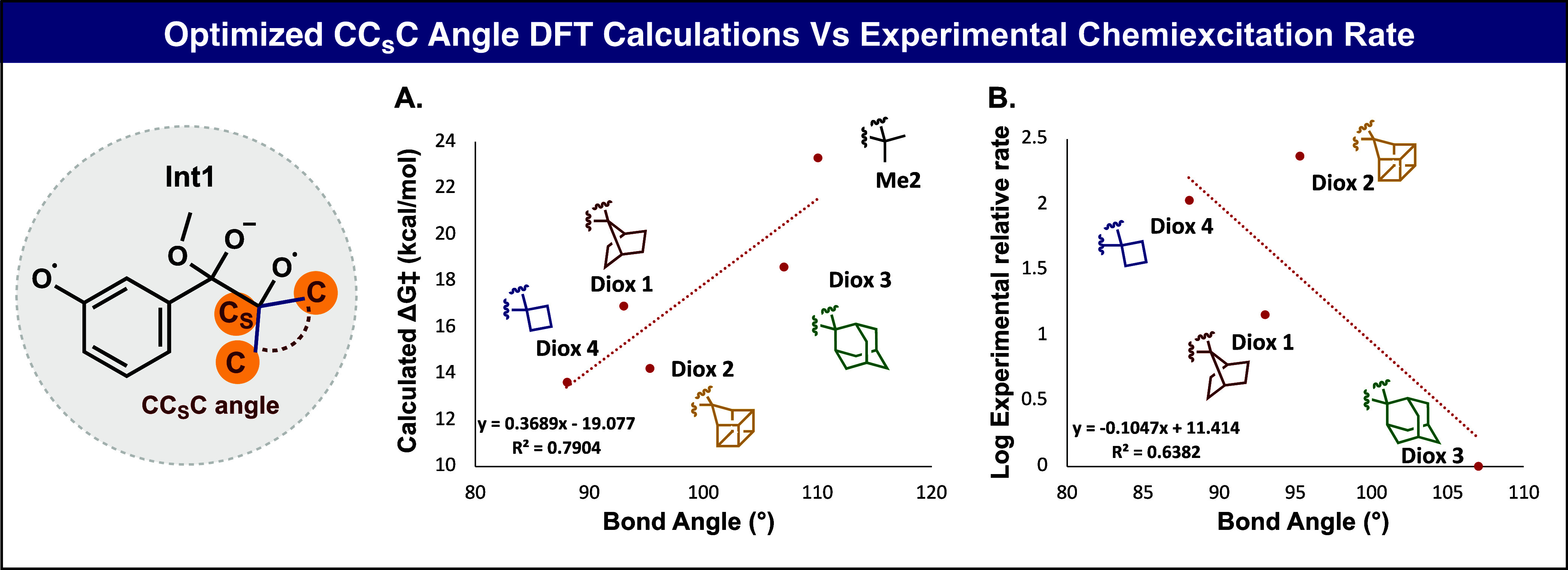
A) Plot of DFT optimized C–Cs–C bond angle in Int1 with calculated free energy barriers. B) Plot of DFT optimized bond angle in Int1 with the logarithm of experimental relative rate.

The last step in synthesizing phenoxy-1,2-dioxetanes involves the oxidation of an enol ether precursor to a dioxetane by singlet oxygen. During this process, a side ene-product can be formed by the elimination of a proton positioned at the allylic position of the enol ether.^39^ We have previously reported that oxidation of cyclopentyl enol ether resulted in the complete formation of the undesired ene-product.^23,24^ On the other hand, the oxidation of the bridged bicyclic and polycyclic enol ethers in this study afforded full conversion to the desired dioxetane product. Here, the formation of an ene-side product is disfavored since the elimination reaction generates a highly constrained poly/bicyclic-alkene. Similarly, oxidation of cyclobutyl and adamantyl enol ethers results in the desired dioxetane with no formation of the ene-side product.

Spiro-cycloalkyl phenoxy-1,2-dioxetanes, which possess enhanced molecular strain, have been demonstrated to undergo accelerated chemiexcitation. One notable example of strain in organic molecules is found in the cubane structure. Cubane is a highly strained hydrocarbon molecule composed of eight carbon atoms, each occupying a corner of a cube, with hydrogen atoms filling in the remaining valences.^27^ The strain in cubane arises from the forced 90-degree bond angles between the carbon atoms, which are significantly smaller than the ideal tetrahedral angle of 109.5 degrees.^40^ Homocubanyl has a small angle of 95 degrees at the spiro-carbon in the dioxetane we have studied and would be expected to be somewhat less reactive than cyclobutyl and 7-norbornyl.^29^ However, the reactivity of homocubanyl is measured to be 16 times that of 7-norbornyl and twice that of the cyclobutyl derivative. There appear to be additional factors beyond the spirostrain accounting for the high reactivity of homocubanyl observed experimentally. Computations also predict high reactivity, although about the same as cyclobutyl which is found experimentally to be one-half as reactive. A possible explanation for this contradictory phenomenon can be found in a study published about 30 years ago by Spitz.^41^ This study showed that the homocubanyl moiety exhibited higher reactivity toward solvolysis reactions compared to a 7-norbornyl counterpart. This reactivity was explained by stabilizing the homocubanyl carbon through two adjacent sigma bonds; the same carbon in this work is referred to as the spiro-carbon (Cs). Relying on the above-mentioned, we suggest that the Cs–O bond of the homocubanyl dioxetane is elongated compared to the cyclobutyl analog, making it more reactive and therefore, increasing its chemiexcitation rate. Despite the high reactivity, the homocubanyl compound can still be isolated and rates of chemiexcitation and decomposition can be measured. This highly strained dioxetane (Diox 2) undergoes extremely fast chemiexcitation upon exposure to fluoride, accompanied by an intense burst of light emission. The attachment of alkyl substituents to the homocubanyl unit, positioned adjacent to the dioxetane ring, could potentially lead to a chemically more stable molecular structure.

The 7-norbornyl unit also possesses a higher degree of angular strain compared to its adamantyl counterpart, but its reactivity is more in line with the spirostrain reflected in the 93° spiro-fusion angle.^29^ This molecule exhibits strain due to its unique structure, with a single methano bridge of the 1 and 4 carbons of a boat cyclohexane. The C1–C7–C4 angle of 93° is consistent with its reactivity, intermediate between adamantyl and cyclobutyl. Indeed, norbornyl is additionally strained by affixing a two-carbon bridge pulling the two ethano linkages in norbornyl toward each other. The strain in the 7-norbornyl-phenoxy-1,2-dioxetane spirofusion leads to a fast chemiexcitation rate and moderate chemical stability in comparison to adamantyl-phenoxy-1,2-dioxetane. This leads to a 12.8-fold increase in the detection sensitivity for a phenoxy-1,2-dioxetane probe spirofused with a 7-norbornyl unit, compared to a probe spirofused with an adamantyl unit.

In summary, we have synthesized and evaluated the chemiluminescence properties of two new spiro-phenoxy-1,2-dioxetanes, fused to bicyclic 7-norbornyl and polycyclic homocubanyl units. The high angular strain of the homocubanyl unit led to an extraordinary chemiexcitation acceleration of the corresponding dioxetane. However, a molecular probe based on spiro-homocubanyl-dioxetane motif was found to be highly unstable. On the other hand, spiro-norbornyl-dioxetane exhibited a substantially higher chemiexcitation rate and moderate chemical stability, when compared to its spiro-adamantyl-dioxetane counterpart. A turn-ON dioxetane probe for the detection of β-gal activity, containing the bicyclic 7-norbornyl unit, exhibited a S/N ratio of 415 in the presence of the enzyme. This probe demonstrated substantially increased detection sensitivity toward β-gal activity in bacteria, with an LOD value that indicates a 12.8-fold increase in sensitivity compared to that obtained by the previously known adamantyl analog. We anticipate that the chemiexcitation acceleration effect of phenoxy-1,2-dioxetane through bridged polycyclic units will create new opportunities for designing innovative chemiluminescence probes with a flash mode of chemiexcitation and increased detection sensitivity.

## Methods

### Stability Assay of Diox 1–Diox 4 (Figure S9 in Appendix II)

Diox 1–Diox 4 stock solutions were prepared in ACN at a final concentration of 10 mM. To a vial containing 270 μL of PBS, 15 μL of ACN and 15 μL of dioxetane stock solution were added and vortexed until a clear solution was obtained. In the case of a slightly cloudy solution, an additional 15 μL ACN was added. The vials were kept at room temperature in the dark. HPLC analysis was conducted for each compound at *T* = 0, 0.5, 4, 8, 16, and 32 h, and the ratio between the percentage of the 1,2-dioxetane and forming benzoate was evaluated at 270 nm by calculating the area under each peak. Three independent measurements were conducted for each dioxetane.

### Chemiluminescent Kinetic Measurements of Diox 1–Diox 4 (Figures S3–S8 in Appendix II)

Diox 1–Diox 4 stock solutions were prepared in ACN at a final concentration of 10 mM. Chemiluminescent kinetic profiles were recorded using Spectramax iD3 with an injector cartridge. The injector settings were fixed on the following parameters: Integration time: 50 ms, injection volume: 10 μL, and measuring interval time: 50 ms. The injectors were prewashed with water, EtOH (70%), and DMSO, and primed with a solution of 100 nM of Diox 1–Diox 4 in ACN before every measurement. Measurements were conducted in a white 96-well Corning plate, each well contained 89 μL DMSO or acetone and 1 μL of TBAF (1 M in THF), with a final volume of 100 μL after the addition via injection of 10 μL of Diox 1–Diox 4 [100 nM] solution. TBAF was added to each well immediately before the beginning of every measurement, and new aliquots were used if measurements were conducted on separate days.

### Visual Demonstrations of the Chemiexcitation for Diox 1–Diox 4 (Figure 3)

Light emission was recorded using a standard camera or iPhone 13Pro. To a vial containing 500 μL of acetone was added 10 μL of TBAF (1 M in THF) and stirred for a few seconds, then 25 μL of Diox 1–Diox 4 was added, and the light emission was recorded for 2 min in total. The recording was taken at 60 to 240 fps speed based on the dioxetane kinetics to allow high sensitivity and yet a small enough file size to analyze. The image sequence was compiled using Adobe Photoshop.

### Chemiluminescent Kinetic Measurements of β-gal Dioxetane Probes (Figures 4 and S10–S16 in Appendix II)

All stock solutions were prepared in ACN at a final concentration of 10 mM. Measurements were recorded by Spectramax iD3 with integration time parameters set at 140 ms. β-gal dioxetane probes were measured in a white 96-well Corning plate, in a final well volume of 100 μL, 1% ACN unless otherwise mentioned. β-galactosidase was added in various concentrations to the well and the light emission was recorded immediately.

## Biological Evaluation (*E. coli* ATCC 25922 Cell Assay)

### Chemiluminescence Measurements of β-gal Dioxetane Probes (Figures 2^4^, S15 and S16)

*E. coli* ATCC 25922 was cultured in LB at 37 °C for 18 h under aerobic conditions. Subsequently, the initial culture was subjected to a PBS wash (centrifuged at 5000 rpm, 10 min), and the bacterial pellet obtained was reconstituted in 4 mL of PBS, aiming for an OD_600_ of 0.8. Following this, a 96-well plate was utilized, and each well was preloaded with 50 μL of the chemiluminescent probes MA-β-gal-norbornyl or MA-β-gal-adamantyl [20 μM, 0.2% ACN]. Next, 50 μL of bacterial aliquot was introduced into each well, bringing the final OD_600_ to 0.4. The resultant chemiluminescence signal was monitored using a Molecular Devices Spectramax iD3 at 37 °C.

### Limit of Detection Measurements for β-gal Dioxetane Probes (Figures 4C2, S15, and S16)

*E. coli* ATCC 25922 was cultured in LB at 37 °C for 18 h under aerobic conditions. Subsequently, the initial culture was subjected to a PBS wash (centrifuged at 5000 rpm, 10 min), and the bacterial pellet obtained was reconstituted in 4 mL of PBS to facilitate a 1:4 dilution experiment. For the subsequent procedure, 96-well plate was utilized, with each well initially loaded with 50 μL of the MA-β-gal-norbornyl or MA-β-gal-adamantyl [20 μM, 0.2% ACN]. Subsequently, 50 μL of bacterial aliquot was introduced into each well, marking the commencement of the 1:4 dilution experiment (which was initiated with an OD_600_ of 0.4). The resultant chemiluminescence signal was monitored using a Molecular Devices Spectramax iD3 at 37 °C.

### Materials

All general reagents, including salts and solvents, were purchased from Sigma-Aldrich, and used as received. Homocubanone (CAS: 15291-18-6) and bicyclo[2.2.1]heptane-7-one (CAS: 10218-02-7) were supplied by Biosynth. Diox 1–Diox 2 and MA-β-gal-norbornyl were prepared as described in the Supporting Information, while the synthesis of Diox 3–Diox 4 and MA-β-gal-adamantyl was referred to in the previous papers.^23,32^ The detailed instrumentation for characterizing synthesized materials and the spectroscopic methods can be found in the Supporting Information.

## Supporting Information Available

Materials and general procedures; detailed experimental and computational methods; characterization of all compounds including ^1^H NMR, ^13^C NMR, HPLC, and MS; additional chemiluminescence and stability measurements (PDF)

## Dedication

In memory of Prof. Philip E. Eaton, a pioneer in the study of the cubane molecular system.
