# Supplementary material for: Boosting Chemiexcitation of Phenoxy-1,2-dioxetanes through 7-Norbornyl and Homocubanyl Spirofusion
Source: JACS Au. 2024 Sep 3;4(9):3558–66. doi: 10.1021/jacsau.4c00493 (PMC11423311; doi:10.1021/jacsau.4c00493)
Supplement: Supplementary file 1 — au4c00493_si_001.pdf [file au4c00493_si_001.pdf]

Supporting Information

Boosting Chemiexcitation of Phenoxy-1,2-Dioxetanes through 7-Norbornyl and Homocubanyl Spirofusion

Sara Gutkin[a#], Omri Shelef[a#], Zuzana Babjaková[b], Laura Anna Tomanová[e], Matej Babjak[e], Tal Kopp[a], Qingyang Zhou[c], Pengchen Ma[c,d], Micha Fridman[a], Urs Spitz[b] ,Kendall N. Houk[c]* and Doron Shabat[a]*

[a]School of Chemistry, Raymond and Beverly Sackler Faculty of Exact Sciences, Tel-Aviv University, Tel Aviv 69978 Israel. [b]BIOSYNTH, Rietlistr. 4 Postfach 125 9422 Staad, Switzerland. [c]Department of Chemistry and Biochemistry, University of California, Los Angeles, California 90095, United States. [d]Department of Chemistry, School of Chemistry, Xi'an Key Laboratory of Sustainable Energy Material Chemistry and Engineering Research Center of Energy Storage Materials and Devices, Ministry of Education, Xi'an Jiaotong University, Xi'an 710049, China. [e]Department of Organic Chemistry, Slovak University of Technology in Bratislava, Radlinskeho 9, 81237 Bratislava, Slovakia.

[#] These authors contributed equally

In memory of Prof. Philip E. Eaton, a pioneer of the Cubane molecular system.

***Corresponding Authors:**

Doron Shabat, Email: chdoron@tauex.tau.ac.il

Kendall N. Houk, Email: houk@chem.ucla.edu

Table of Contents

General methods S3

Synthesis and characterization S4-S15

Appendix I - Computational data S16-S24

Appendix II - Supplementary figures S25-S34

Appendix III – Spectral data of compounds S35-S42

References S43-S44

General methods

All reactions requiring anhydrous conditions were performed under an Argon atmosphere. All reactions were carried out at room temperature unless stated otherwise. Chemicals and solvents were either A.R. grade or purified by standard techniques. Thin-layer chromatography (TLC): silica gel plates Merck 60 F254: compounds were visualized by irradiation with UV light. Column chromatography (FC): silica gel Merck 60 (particle size 0.040-0.063 mm), eluent given in parentheses. Reverse-phase high-pressure liquid chromatography (RP-HPLC): C18 5u, 250x4.6mm, eluent given in parentheses. Preparative RP-HPLC: C18 5u, 250x21mm, eluent given in parentheses. ^{1}H-NMR spectra were measured using Bruker Avance operated at 400MHz. ^{13}C-NMR spectra were measured using Bruker Avance operated at 101 MHz. Chemical shifts were reported in ppm on the δ scale relative to a residual solvent ($CDCl_3$: δ = 7.26 for ^{1}H-NMR and 77.16 for ^{13}C-NMR, DMSO-d_6: δ = 2.50 for ^{1}H-NMR and 39.52 for ^{13}C-NMR and ACN-d_3: δ = 2.04 for ^{1}H-NMR and 29.8 for ^{13}C-NMR). Mass spectra were measured on Waters Xevo TQD. Chemiluminescence was recorded on Molecular Devices Spectramax iD3. Fluorescence was recorded on Tecan Infinite 200 Pro. All general reagents, including salts and solvents, were purchased from Sigma-Aldrich. Homocubanone (CAS: 15291-18-6) and Bicyclo[2.2.1]heptane-7-one (CAS: 10218-02-7) were supplied by Biosynth®. Light irradiation for photochemical reactions: LED PAR38 lamp (19W, 3000K).

Abbreviations

ACN- Acetonitrile, **$CHCl_3$**- Chloroform, **DCM**- Dichloromethane, **DMF**- N,N'–Dimethylformamide, **EtOAc**- Ethyl acetate, **Et_3N**– Triethylamine, **Hex**- Hexanes, **LDA**- Lithium diisopropylamide, **MB**- Methylene blue, **MeOH**- Methanol, **$MgCl_2$-** Magnesium chloride, **$P(OMe)_3$**- Trimethyl phosphite, **TBAF**– Tetra-n-butylammonium fluoride, **TBS**– Tert-butyldimethylsilyl, **THF**- Tetrahydrofuran, **TMS-Cl**- Trimethylsilyl chloride, **TFA** - Trifluoroacetic acid.

Synthesis and Characterization

General synthetic procedures for the formation of TBS-masked Diox 1-4

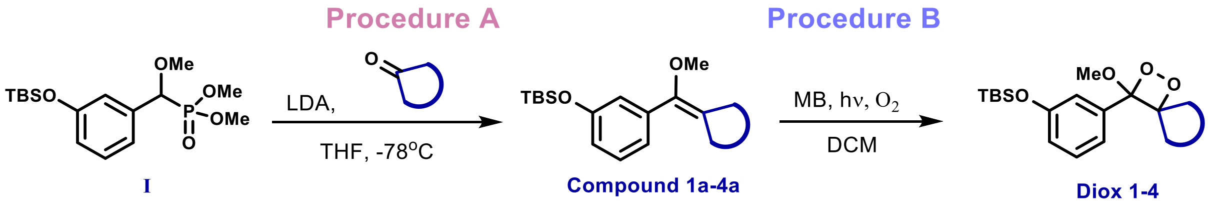

Diox 3 and **Diox 4** were synthesized according to a known procedure.[1,2] All compounds were synthesized according to general **procedures A** and **B**.

***Procedure A** - Wittig-Horner reaction*

Phosphonate I[1] (1.0 equiv.) was dissolved in anhydrous THF under argon atmosphere at -78°C. LDA (1.2 equiv.) was added dropwise, and the solution was stirred for 20 minutes. Cycloalkyl ketone (1.2 equiv.) was added, and after 10 minutes of stirring at -78°C, the reaction was warmed to room temperature and stirred for additional 20 minutes. The reaction was monitored by TLC (Hex:EtOAc mixture). Upon completion, the reaction mixture was diluted with EtOAc and washed with brine. The organic phase was dried over Na_2SO_4, filtered, and concentrated under reduced pressure. Purification of the crude residues by column chromatography (Hex:EtOAc mixture) afforded the desired enolethers (compound **1a-2a**).

***Procedure B** – Oxidation reaction*

Enolethers (Compound 1a-4a) (~0.02-0.1 mmol) were dissolved in 5 mL of DCM followed by the addition of a catalytic amount of methylene blue. The solution was cooled to 0°C. Then, oxygen was bubbled through the solution while irradiating with yellow light. The reaction was monitored by TLC (Hex:EtOAc mixture) or RP-HPLC (gradient of 90-100% ACN in water, 0.1% TFA). Upon completion (about 5 min) the crude product was immediately passed through a silica gel column (Hex:EtOAc mixture) to filter out methylene blue. The solvents were removed under reduced pressure while cooling the bath to 10°C. If needed, the compounds were further purified by RP-HPLC using 100% ACN as an eluent to obtain **Diox 1** and **Diox 2**.

Oxidation reaction setup:

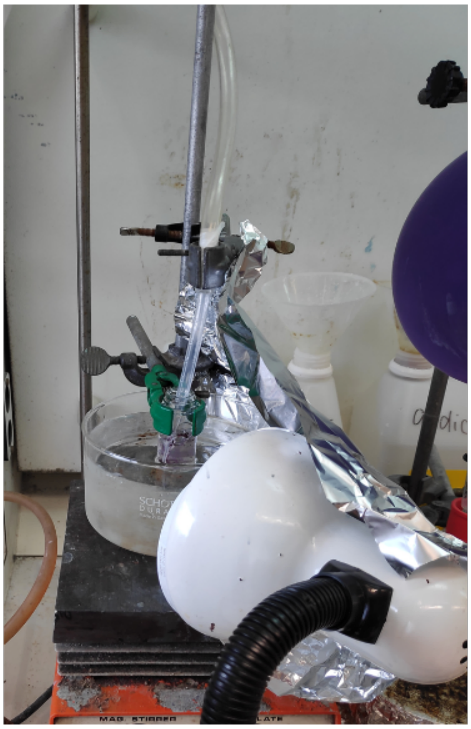

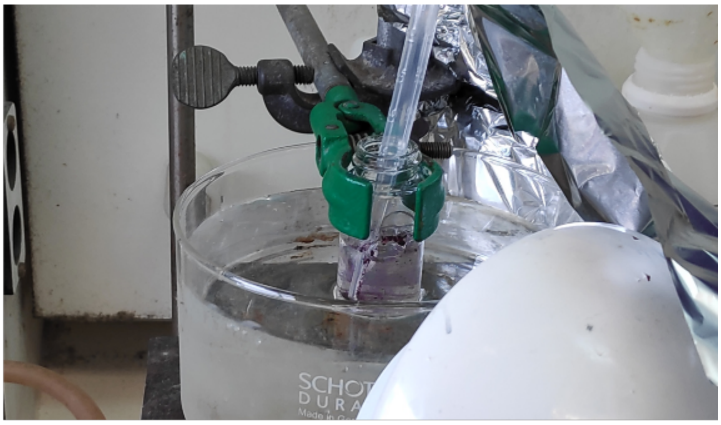

Oxidation setup:

Cooling bath
Lamp
Oxygen (cylinder)

The reaction setup consists of a cooling bath, a lamp, and an oxygen cylinder. The reaction vial was cooled in an ice water bath and oxygen was vigorously bubbled through while irradiating with white light for 8 minutes. The solvent was evaporated without heating the water bath and filtered through a short pad of silica.

Synthesis and characterization of Diox 1 and Diox-2

Bicyclic derivative

Oxidation of all Bicyclic and polycyclic enolether derivatives (Compounds **1a** and **2a**) yielded no ene-product and afforded only the 1,2-dioxetane analogs.

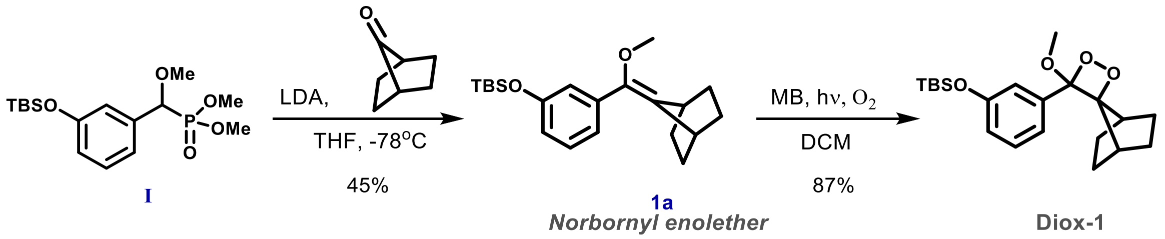

Norbornyl enolether (Compound 1a)

Compound **1a** was synthesized according to **Procedure A**, using phosphonate **I**[1] (300 mg, 0.83 mmol) in dry THF (1.5 mL), LDA (2.0M in THF, 0.5mL, 1.00 mmol) and Bicyclo[2.2.1]heptane-7-one (90 mg, 1.00 mmol). The crude residue was purified by column chromatography (90:10, Hex:EtOAc) to afford compound **1a** in the form of a yellow oil (120 mg, mmol, 45%).

^{1}H-NMR (400 MHz, $CDCl_3$) δ 7.21 (dd, J = 10.0, 5.7 Hz, 1H), 7.03 – 6.98 (m, 1H), 6.91 – 6.89 (m, 1H), 6.79 (ddd, J = 8.0, 2.5, 1.0 Hz, 1H), 3.46 (s, 3H), 3.04 (t, J = 3.5 Hz, 1H), 2.71 (d, J = 3.4 Hz, 1H), 1.79 – 1.65 (m, 4H), 1.47 – 1.37 (m, 4H), 1.03 (s, 9H), 0.24 (s, 6H).

^{13}C-NMR (101 MHz, $CDCl_3$) δ 155.41, 140.07, 137.57, 134.70, 128.93, 121.53, 119.90, 119.25, 58.70, 37.26, 36.02, 29.56, 29.32, 25.72, 18.23, -4.36.

3D HPLC spectra (90-100% ACN in water, 0.1%TFA)

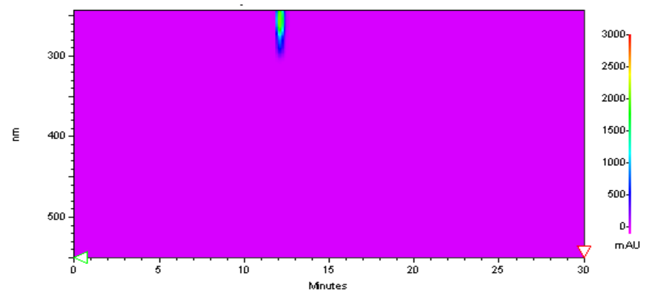

2D HPLC spectra (Absorbance measured at 303nm)

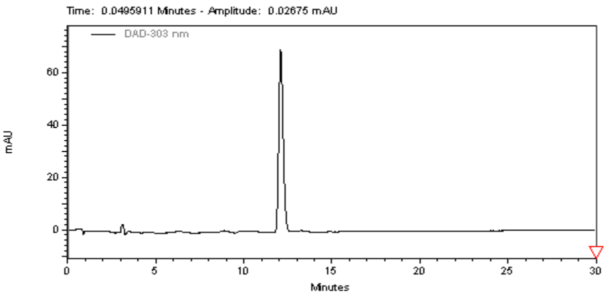

Diox 1

Compound **1a** (15 mg, 0.04 mmol) was reacted according to **Procedure B**. Reaction was monitored by RP-HPLC (90-100% ACN in water, 0.1%TFA). Upon completion (5 min), the crude product was purified by column chromatography (90:10, Hex:EtOAc) to afford **Diox 1** in the form of a yellow oil (14 mg, 87% yield).

^{1}H-NMR (400 MHz, $CDCl_3$) δ 7.28 (dd, J = 13.9, 6.1 Hz, 1H), 7.24 (d, J = 7.7 Hz, 1H), 7.11 (s, 1H), 6.88 (ddd, J = 7.7, 2.5, 1.4 Hz, 1H), 3.29 (s, 3H), 2.65 (t, J = 4.0 Hz, 1H), 1.92 (t, J = 3.7 Hz, 1H), 1.82 – 1.63 (m, 4H), 1.30 – 1.15 (m, 4H), 0.98 (s, J = 3.0 Hz, 9H), 0.20 (s, 6H).

^{13}C-NMR (101 MHz, $CDCl_3$) δ 156.08, 136.69, 129.68, 121.42, 120.82, 119.35, 111.44, 105.02, 50.49, 39.13, 37.79, 26.28, 25.94, 25.80, 25.51, 24.77, 18.34, -4.27.

3D HPLC spectra (90-100% ACN in water, 0.1%TFA)

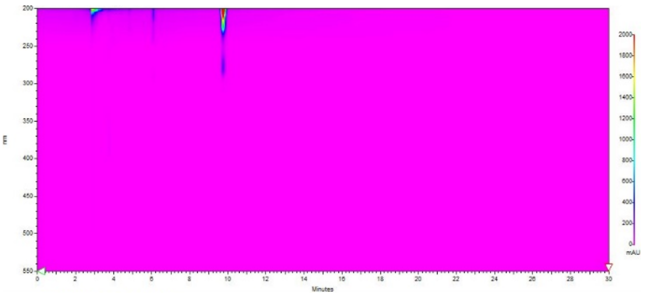

2D HPLC spectra (Absorbance measured at 303nm)

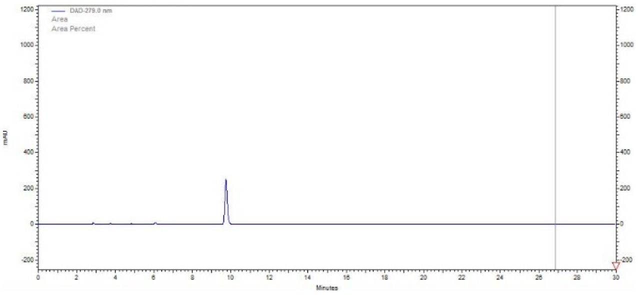

Polycyclic derivative

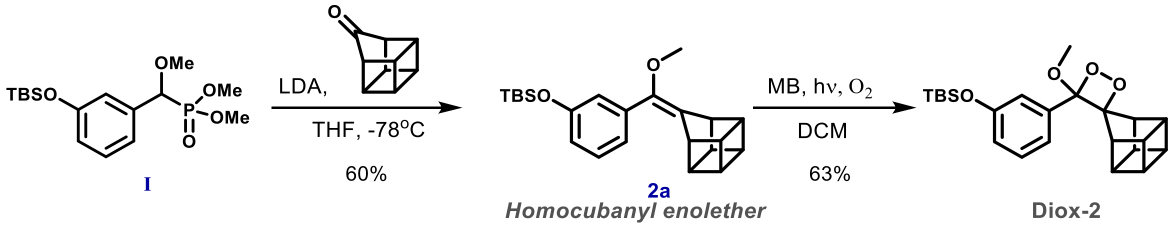

Compound 2a

Compound **2a** was synthesized according to **Procedure A**, using phosphonate I[1] (300 mg, 0.83 mmol) in dry THF (1.5 mL), LDA (2.0M in THF, 0.5mL, 1.00 mmol), and Homocubanone (110 mg, 1.00 mmol). The crude residue was purified by column chromatography (90:10, Hex:EtOAc) to afford compound **2a** in the form of a yellow oil (175 mg, mmol, 60%).

^{1}H-NMR (400 MHz, CD_3CN) δ 6.99 (dd, J = 8.7, 7.7 Hz, 1H), 6.75 – 6.69 (m, 1H), 6.60 – 6.55 (m, 2H), 3.75 – 3.71 (m, 1H), 3.38 (dd, J = 5.2, 3.9 Hz, 1H), 3.26 – 3.18 (m, 3H), 3.14 (s, 3H), 2.98 – 2.88 (m, 2H), 1.70 (dt, J = 4.9, 2.5 Hz, 1H), 0.74 (s, J = 3.0 Hz, 9H), -0.03 (s, 6H).

^{13}C-NMR (101 MHz, CD_3CN) δ 155.58, 141.32, 139.93, 137.50, 129.39, 121.89, 120.17, 119.80, 117.45, 58.34, 44.86, 44.76, 44.09, 42.58, 41.32, 41.18, 25.16, 18.01, -5.06.

3D HPLC spectra (90-100% ACN in water, 0.1%TFA)

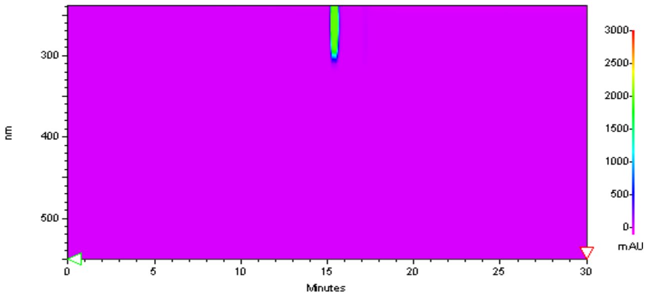

2D HPLC spectra (Absorbance measured at 303nm)

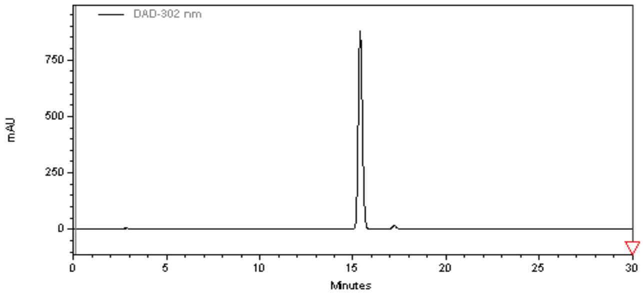

Diox 2

Compound **2a** (15 mg, 0.04 mmol) was reacted following **Procedure B**. Reaction was monitored by RP-HPLC (90-100% ACN in water, 0.1%TFA). Upon completion (15 min), the crude product was purified by column chromatography (90:10, Hex:EtOAc) to afford **Diox 2** in the form of yellow oil (10 mg, 63 % yield).

^{1}H-NMR (400 MHz, CD_3CN) δ 7.36 (dd, J = 11.9, 4.1 Hz, 1H), 7.09 (ddd, J = 7.7, 1.5, 1.1 Hz, 1H), 6.96 (ddd, J = 8.1, 2.5, 1.0 Hz, 1H), 6.92 – 6.90 (m, 1H), 3.73 (dd, J = 7.5, 2.9 Hz, 1H), 3.54 – 3.45 (m, 3H), 3.29 – 3.23 (m, 1H), 3.15 (s, 3H), 3.12 (dtd, J = 4.8, 3.3, 2.0 Hz, 1H), 3.01 – 2.95 (m, 1H), 2.84 (ddd, J = 6.6, 4.3, 1.6 Hz, 1H), 1.96 (dt, J = 4.9, 2.5 Hz, 2H), 1.01 (s, 9H), 0.23 (s, 6H). **NMR has 15% of dioxetane decomposition to benzoate, indicative peak of methyl ester at: 3.87 ppm.*

^{13}C-NMR (101 MHz, CD_3CN) δ 166.57, 156.09, 155.89, 137.37, 131.89, 130.04, 129.92, 124.95, 122.51, 121.27, 120.72, 119.85, 118.04, 117.43, 51.89, 49.90, 46.69, 45.60, 44.48, 43.81, 42.49, 41.83, 39.05, 25.15, -5.17.

3D HPLC spectra (90-100% ACN in water, 0.1%TFA)

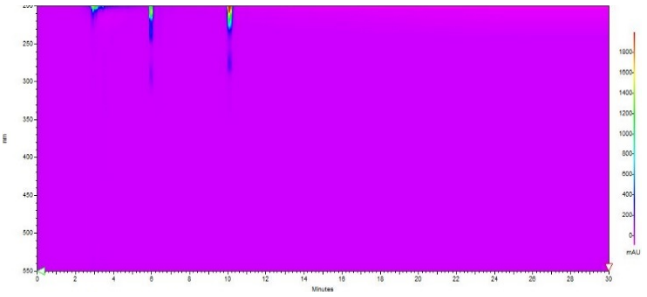

2D HPLC spectra (Absorbance measured at 303nm)

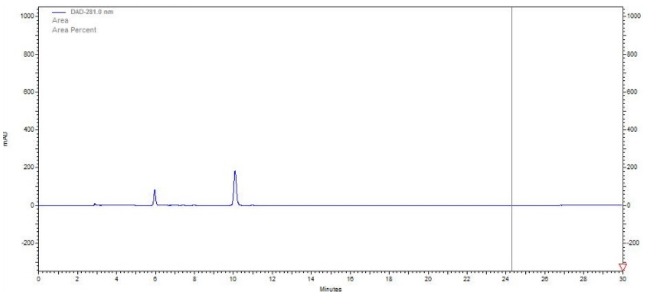

**HPLC has 15% of dioxetane decomposition to benzoate, retention time of 6 minutes.*

Synthetic procedures for the β-gal-masked-acrylate 1,2-dioxetanes

Synthesis of norbornyl and cyclobutyl-1,2-dioxetanes

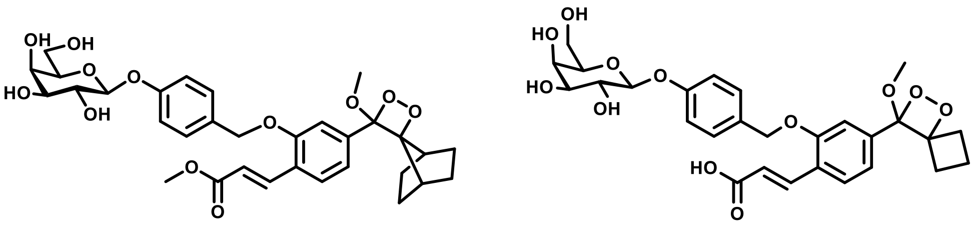

MA- -gal-norbornyl MA- -gal-cyclobutyl

Probe MA-β-gal-adamantyl was synthesized according to a known procedure.[1]

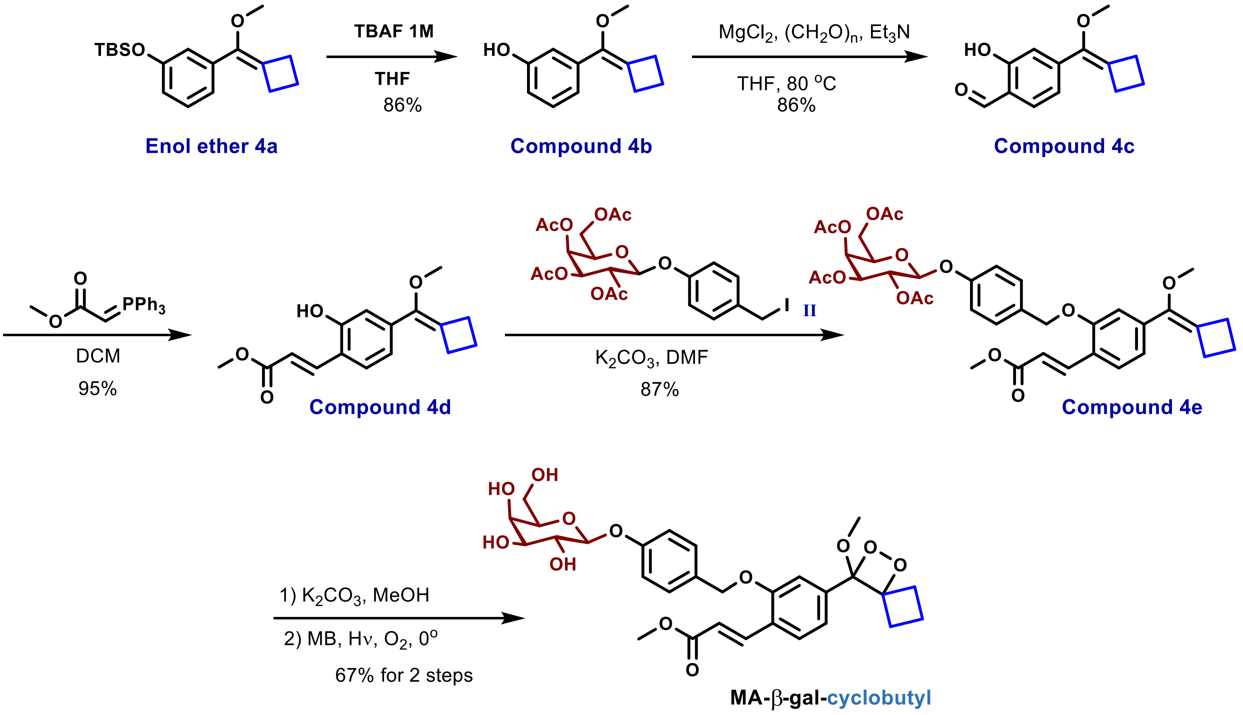

Enol ether 4a was synthesized according to a known procedure.[2]

Compound 4b

TBAF (1M, 660 µL, 0.66 mmol) was added into a solution of compound **4a** (200 mg, 0.66 mmol) in THF (4 mL). The reaction was stirred at room temperature and was monitored by TLC (90:10, Hex:EtOAc). Upon completion, the reaction mixture was diluted with EtOAc and washed with 1M HCl. The organic layer was separated, washed with brine, dried over Na_2SO_4, and evaporated under reduced pressure. The residue was purified by column chromatography (90:10, Hex:EtOAc) to affored compound **4b** in the form of a pale-yellow oil (110 mg, 0.56 mmol, 86% yield).

^{1}H-NMR (400 MHz, $CDCl_3$) δ 7.19 (t, J = 8.0 Hz, 1H), 6.91 (t, J = 4.2 Hz, 2H), 6.76 – 6.70 (m, 1H), 3.58 (s, 3H), 2.94 (t, J = 7.8 Hz, 4H), 2.10 – 1.99 (m, 2H).

^{13}C-NMR (101 MHz, $CDCl_3$) δ 156.04, 145.75, 136.81, 127.0, 118.80, 114.20, 112.82, 60.91, 58.87, 52.54, 30.84, 28.96, 25.75, 18.15, 14.28.

MS (ES-): *m/z* calc. $C_{12}H_{14}O_2$:190.1; found: 189.2 $[M-H]^-$.

Compound 4c

Et_3N (510 µL, 3.67 mmol) was added into a solution of compound **4b** (200 mg, 1.05 mmol) in dry THF (4 mL). Then, $MgCl_2$ (174 mg, 1.83 mmol) and paraformaldehyde (227 mg, 7.33 mmol) were added. The reaction was heated under reflux and was monitored by TLC (90:10, Hex:EtOAc). Upon completion, the reaction mixture was diluted with EtOAc and washed with 1M HCl. The organic layer was separated, washed with brine, dried over Na_2SO_4, and evaporated under reduced pressure. The residue was purified by column chromatography (90:10, Hex:EtOAc) affording compound **4c** in the form of a pale-yellow oil (167 mg, 0.76 mmol, 86% yield).

^{1}H-NMR (400 MHz, $CDCl_3$) δ 11.03 (s, 1H), 9.80 (s, 1H), 7.46 (d, J = 8.1 Hz, 1H), 6.99 (dd, J = 8.2, 1.5 Hz, 1H), 6.94 (s, 1H), 3.56 (s, 3H), 3.05 – 2.90 (m, 4H), 2.08 (quin, J = 7.9 Hz, 2H).

^{13}C-NMR (101 MHz, $CDCl_3$) δ 195.77, 161.82, 145.58, 144.48, 133.63, 132.45, 119.26, 117.66, 114.32, 59.12, 31.74, 31.52, 29.82, 29.57, 18.18.

MS (ES-): *m/z* calc. $C_{13}H_{14}O_3$: 218.9; found: 217.2 $[M-H]^-$.

Compound 4d

To a solution of compound **4c** (100 mg, 0.46 mmol) in DCM (1 mL), methyl (triphenyl-phosphoranylidene) acetate (150 mg, 0.46 mmol) was added. The reaction was stirred for 15 min and monitored by TLC (80:20, Hex:EtOAc). Upon completion, the reaction mixture was diluted with DCM and washed with 1M HCl followed by an additional wash with brine. The organic layer was then dried over Na_2SO_4, filtered, and concentrated under reduced pressure. The crude residue was purified by column chromatography (80:20, Hex:EtOAc) to obtain compound **4d** in the form of a white solid (120 mg, 0.43 mmol, 95%).

^{1}H-NMR (400 MHz, DMSO) δ 10.61 (s, 1H), 7.84 (d, J = 16.2 Hz, 1H), 7.72 (d, J = 8.1 Hz, 1H), 7.40 (d, J = 1.5 Hz, 1H), 7.30 (dd, J = 8.1, 1.4 Hz, 1H), 6.72 (d, J = 16.2 Hz, 1H), 3.70 (s, J = 4.3 Hz, 3H), 3.31 (s, 3H), 2.24 – 2.16 (m, 4H), 2.04 – 1.98 (m, 1H), 1.82 – 1.72 (m, 1H).

^{13}C-NMR (101 MHz, DMSO) δ 200.31, 167.44, 157.35, 139.45, 137.84, 129.76, 125.56, 119.75, 118.62, 115.78, 60.33, 55.47, 52.06, 42.19, 29.61, 25.08, 21.32, 18.12, 14.64.

MS (ES-): *m/z* calc. $C_{16}H_{18}O_4$:274.1; found: 273.3 $[M-H]^-$.

Compound 4e

Compound **4d** (50 mg, 0.18 mmol) was dissolved in dry DMF (1 mL). K_2CO_3 (37 mg, 0.48 mmol) was added, and the solution was stirred for 10 minutes at room temperature before compound **II**[1] (130 mg, 0.27 mmol) was

added. The reaction mixture was stirred for 1 hour and monitored by TLC (50:50, Hex:EtOAc). After completion, the reaction mixture was diluted with EtOAc and washed with saturated 1M HCl. The organic layer was separated, washed with brine, dried over Na_2SO_4, and evaporated under reduced pressure. The crude product was purified by column chromatography (50:50, Hex:EtOAc) to obtain compound **2e** in the form of a white solid (90 mg, 0.14 mmol, 87%) .

^{1}H-NMR (400 MHz, $CDCl_3$) δ 8.00 (d, J = 16.2 Hz, 1H), 7.47 (dd, J = 11.8, 7.5 Hz, 1H), 7.35 (d, J = 8.7 Hz, 2H), 7.04 – 6.97 (m, 2H), 6.93 (dd, J = 4.0, 2.8 Hz, 1H), 6.49 (d, J = 16.1 Hz, 1H), 5.51 – 5.42 (m, 2H), 5.16 – 5.01 (m, 4H), 4.18 (qd, J = 11.3, 6.6 Hz, 2H), 4.06 (t, J = 6.6 Hz, 1H), 3.76 (s, 3H), 3.51 (s, 3H), 2.97 – 2.83 (m, 4H), 2.17 (s, 3H), 2.06 (s, 3H), 2.04 (s, 3H), 2.00 (s, 3H).

^{13}C-NMR (101 MHz, $CDCl_3$) δ 170.51, 170.38, 170.25, 169.56, 168.14, 157.35, 156.84, 145.91, 139.93, 138.96, 131.67, 129.08, 128.94, 128.73, 128.41, 122.28, 119.09, 117.86, 117.25, 110.08, 99.79, 71.19, 70.96, 69.93, 68.74, 67.01, 61.49, 58.94, 51.69, 31.11, 29.74, 29.29, 20.86, 20.78, 20.71, 18.21.

MS (ES+): *m/z* calc. $C_{37}H_{42}O_{14}$: 710.2; found: 733.7 $[M+Na]^+$.

Probe MA-β-gal-cyclobutyl

Compound **2e** (50 mg, 0.07 mmol) was dissolved in MeOH (1 mL). K_2CO_3 (97 mg, 0.70 mmol) was added, and the solution was stirred at room temperature for 30 minutes. The reaction was monitored by RP-HPLC (30-100% ACN, ammonium carbonate buffer [30 mM]). Upon completion, the reaction mixture was diluted with EtOAc and washed with brine. The organic phase was dried over Na_2SO_4, filtered, and concentrated under reduced pressure. The crude product was further reacted without purification. The crude product and catalytic amount of methylene blue (~1 mg) were dissolved in 10 mL of DCM and cooled to 0°C. Oxygen was bubbled through the solution while irradiating with yellow light. The reaction was monitored by RP-HPLC (30-100% ACN, ammonium carbonate buffer [30 mM]). After completion, the reaction mixture was concentrated by evaporation under reduced pressure at 10°C. The crude product was purified by preparative RP-HPLC (30-100% ACN, ammonium carbonate buffer [30 mM]) to afford **Probe MA-β-gal-CB** in the form of a white solid (5 mg, 67% yield).

^{1}H-NMR (400 MHz, CD_3CN) δ 7.98 – 7.91 (m, 1H), 7.73 (t, J = 6.3 Hz, 2H), 7.44 – 7.38 (m, 2H), 7.17 (dt, J = 12.2, 6.5 Hz, 2H), 7.10 (qd, J = 5.1, 2.6 Hz, 2H), 6.62 (dd, J = 16.2, 5.0 Hz, 1H), 5.23 – 5.14 (m, 2H), 4.87 (dd, J = 9.3, 7.6 Hz, 1H), 3.86 (s, 1H), 3.73 (d, J = 1.2 Hz, 3H), 3.67 (ddd, J = 12.2, 6.4, 3.6 Hz, 3H), 3.53 (d, J = 11.4 Hz, 2H), 3.31 (d, J = 3.3 Hz, 1H), 3.27 (d, J = 0.8 Hz, 3H), 3.12 (dd, J = 12.5, 6.6 Hz, 1H), 3.02 (t, J = 8.2 Hz, 1H), 2.97 – 2.90 (m, 1H), 2.22 – 2.07 (m, 2H), 1.79 – 1.65 (m, 1H), 1.60 – 1.50 (m, 1H), 1.30 – 1.15 (m, 1H).

^{13}C-NMR (101 MHz, CD_3CN) δ 167.42, 157.69, 138.96, 138.82, 138.40, 130.44, 129.70, 129.59, 129.42, 128.93, 124.53, 121.90, 120.77, 119.53, 119.29, 117.52, 113.49, 111.74, 111.45, 101.15, 94.67, 75.38, 73.44, 71.20, 70.33, 70.24, 68.94, 61.40, 52.16, 51.49, 51.40, 50.53, 47.29, 32.97, 30.95, 10.87.

MS (ES+): *m/z* calc. $C_{29}H_{34}O_{12}$: 574.2; found: 597.6 $[M+Na]^+$.

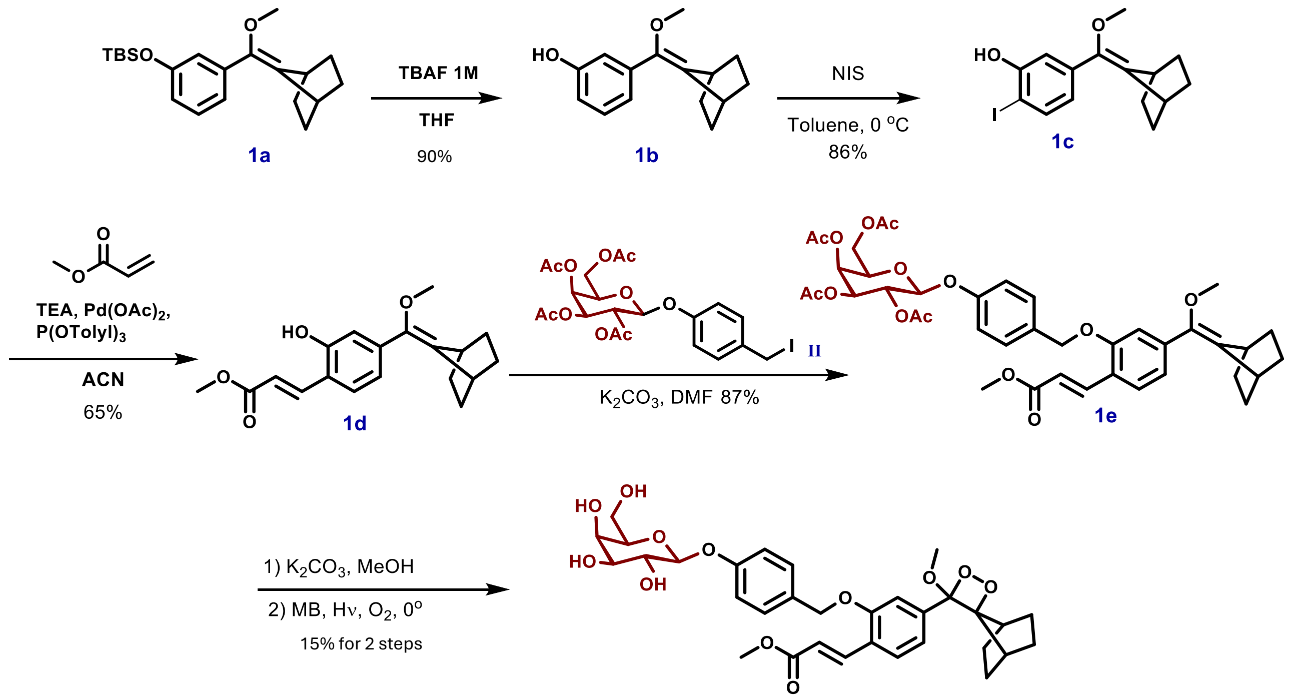

MA-β-gal-NORB

Compound 1b

TBAF (1M, 581 µL, 0.58 mmol) was added into a solution of compound **1a** (200 mg, 0.58 mmol) in THF (4 mL). The reaction was stirred at room temperature and was monitored by TLC (90:10, Hex:EtOAc). Upon completion, the reaction mixture was diluted with EtOAc and washed with 1M HCl. The organic layer was separated, washed with brine, dried over Na_2SO_4, and evaporated under reduced pressure. The residue was purified by column chromatography (90:10, Hex:EtOAc) affording compound **1b** in the form of a pale-yellow oil (120 mg, 0.52 mmol, 90% yield).

^{1}H-NMR (400 MHz, $CDCl_3$) δ 7.18 (dd, J = 11.5, 4.7 Hz, 1H), 6.96 – 6.90 (m, 2H), 6.79 – 6.75 (m, 1H), 3.46 (s, 3H), 3.00 (s, 1H), 2.71 (s, 1H), 1.68 (d, J = 3.2 Hz, 4H), 1.46 – 1.32 (m, 5H).

^{13}C-NMR (101 MHz, $CDCl_3$) δ 156.00, 139.70, 137.62, 135.88, 129.36, 120.90, 115.03, 114.76, 59.01, 37.41, 36.30, 29.63, 29.36.

MS (ES-): *m/z* calc. $C_{15}H_{18}O_2$:230.1; found: 229.2 $[M-H]^-$.

Compound 1c

Compound **1b** (120 mg, 0.52 mmol) was dissolved in Toluene and cooled to 0°C. N-Iodosuccinimide (510 µL, 3.67 mmol) was added portion wise into the solution and the reaction was monitored by TLC (90:10, Hex:EtOAc). Upon completion, the reaction mixture was diluted with EtOAc and washed with saturated solution of sodium thiosulphate. The organic layer was separated, washed with brine, dried over Na_2SO_4, and evaporated under reduced pressure. The residue was purified by column chromatography (90:10, Hex:EtOAc) affording compound **3c** in the form of a pale-yellow oil (160 mg, 0.44 mmol, 86% yield).

^{1}H-NMR (400 MHz, CDCl$_3$) δ 7.60 (d, J = 8.2 Hz, 1H), 7.02 (d, J = 2.0 Hz, 1H), 6.71 (dd, J = 8.2, 2.0 Hz, 1H), 3.44 (s, 3H), 3.00 (d, J = 3.5 Hz, 1H), 2.68 (d, J = 2.8 Hz, 1H), 1.75 – 1.62 (m, 4H), 1.40 (dd, J = 6.1, 3.7 Hz, 5H).

^{13}C-NMR (101 MHz, CDCl$_3$) δ 154.80, 139.16, 138.87, 137.94, 136.47, 122.60, 114.82, 84.12, 59.15, 46.19, 37.44, 36.33, 31.76, 30.49, 29.83, 29.61, 25.13.

MS (ES-): *m/z* calc. $C_{15}H_{17}IO_2$: 356.0; found: 355.2 $[M\text{-}H]^-$.

Compound 1d

Compound **1c** (160 mg, 0.44 mmol), methyl acrylate (3eq), and Et3N (1.5eq) were dissolved in anhydrous ACN. Then Pd(OAc)2 (0.05eq) and P(o-tol)3 (0.01eq) were added. The flask was sealed, and the solution was stirred at 120°C for 2 hours. Reaction was monitored by TLC (Hex:EtOAc 80:20). Upon completion, the reaction mixture was diluted with EtOAc and washed with saturated NH_4Cl. The organic phase was dried over Na_2SO_4 and concentrated under reduced pressure. The residue was purified by column chromatography on silica gel (Hex:EtOAc 85:15) to afford the compound **1d** in the form of a white solid (90 mg, 0.28 mmol, 65%).

^{1}H-NMR (400 MHz, CD$_3$OD) δ 7.95 (d, J = 16.1 Hz, 1H), 7.47 (d, J = 8.0 Hz, 1H), 6.90 – 6.84 (m, 2H), 6.60 (d, J = 16.1 Hz, 1H), 3.77 (s, 3H), 3.43 (s, 3H), 3.30 (dt, J = 3.1, 1.5 Hz, 1H), 2.97 (s, 1H), 2.72 (s, 1H), 1.74 – 1.60 (m, 4H), 1.47 – 1.38 (m, 5H).

^{13}C-NMR (101 MHz, CD$_3$OD) δ 168.75, 156.87, 140.66, 140.06, 139.60, 136.14, 128.60, 120.60, 119.49, 116.61, 115.20, 58.08, 50.75, 37.29, 36.13, 29.13, 28.80.

MS (ES-): *m/z* calc. $C_{19}H_{22}O_4$:314.1; found: 313.3 $[M\text{-}H]^-$.

Compound 1e

Compound **1d** (90 mg, 0.28 mmol) was dissolved in dry DMF (1 mL). K_2CO_3 (116 mg, 0.84 mmol) was added, and the solution was stirred for 10 minutes at room temperature before compound **VIII**[1] (130 mg, 0.27 mmol) was added. The reaction mixture was stirred for 1 hour and monitored by TLC (50:50, Hex:EtOAc). After completion, the reaction mixture was diluted with EtOAc and washed with saturated 1M HCl. The organic layer was separated, washed with brine, dried over Na_2SO_4, and evaporated under reduced pressure. The crude product was purified by column chromatography (50:50, Hex:EtOAc) to obtain compound **3e** in the form of a white solid (180 mg, 0.24 mmol, 87%) .

^{1}H-NMR (400 MHz, CDCl$_3$) δ 8.03 (d, J = 16.2 Hz, 1H), 7.48 (d, J = 8.0 Hz, 1H), 7.35 (d, J = 8.5 Hz, 2H), 7.03 – 6.91 (m, 4H), 6.52 (d, J = 16.1 Hz, 1H), 5.51 – 5.42 (m, 2H), 5.14 – 5.07 (m, 3H), 5.03 (d, J = 8.0 Hz, 1H), 4.19 (qd, J = 11.3, 6.6 Hz, 2H), 4.06 (t, J = 6.6 Hz, 1H), 3.78 (s, 3H), 3.38 (s, 3H), 3.03 – 2.92 (m, 1H), 2.54 (d, J = 3.7 Hz, 1H), 2.18 (s, 3H), 2.06 (s, 3H), 2.04 (s, 3H), 2.01 (s, 3H), 1.73 – 1.56 (m, 4H), 1.44 – 1.34 (m, 4H).

^{13}C-NMR (101 MHz, CDCl$_3$) δ 170.50, 170.39, 170.27, 169.54, 168.11, 157.10, 156.86, 139.94, 139.79, 137.04, 131.63, 128.89, 128.57, 122.90, 121.23, 118.11, 117.26, 112.41, 99.81, 71.20, 70.97, 69.90, 68.74, 67.00, 61.49, 59.15, 51.73, 37.40, 36.38, 29.65, 29.23, 20.87, 20.79, 20.72.

MS (ES+): *m/z* calc. $C_{40}H_{46}O_{14}$: 750.2; found: 789.8 $[M+K]^+$.

Probe MA-β-gal-Norbornyl

Compound **1e** (50 mg, 0.08 mmol) was dissolved in MeOH (1 mL). K_2CO_3 (97 mg, 0.80 mmol) was added, and the solution was stirred at room temperature for 30 minutes. The reaction was monitored by RP-HPLC (30-100% ACN, ammonium carbonate buffer [30 mM]). Upon completion, the reaction mixture was diluted with EtOAc and washed with brine. The organic phase was dried over Na_2SO_4, filtered, and concentrated under reduced pressure. The crude product was further reacted without purification. The crude product and catalytic amount of methylene blue (~1 mg) were dissolved in 10 mL of DCM and cooled to 0°C. Oxygen was bubbled through the solution while irradiating with yellow light. The reaction was monitored by RP-HPLC (30-100% ACN, ammonium carbonate buffer [30 mM]). After completion, the reaction mixture was concentrated by evaporation under reduced pressure at 10°C. The crude product was purified by preparative RP-HPLC (30-100% ACN, ammonium carbonate buffer [30 mM]) to afford **Probe MA-β-gal-Norbornyl** in the form of a white solid (3 mg,15% yield).

^{1}H-NMR (400 MHz, CD_3OD) δ 8.05 (d, J = 16.2 Hz, 1H), 7.71 (d, J = 8.0 Hz, 1H), 7.38 (d, J = 8.7 Hz, 2H), 7.27 (dd, J = 6.7, 5.5 Hz, 2H), 7.12 (dd, J = 8.7, 0.9 Hz, 2H), 6.67 (d, J = 16.2 Hz, 1H), 5.31 – 5.17 (m, 2H), 3.94 – 3.90 (m, 1H), 3.83 – 3.75 (m, 7H), 3.71 – 3.67 (m, 1H), 3.59 (dd, J = 9.7, 3.4 Hz, 1H), 3.24 (s, 3H), 2.55 (d, J = 3.5 Hz, 1H), 1.64 (d, J = 1.3 Hz, 5H), 1.21 (ddd, J = 21.6, 18.9, 9.3 Hz, 4H), 0.93 – 0.69 (m, 2H).

^{13}C-NMR (101 MHz, CD_3OD) δ 167.96, 157.73, 157.05, 139.16, 138.54, 130.26, 128.70, 128.52, 124.60, 119.94, 118.84, 116.59, 112.37, 110.74, 104.55, 101.50, 75.52, 73.44, 70.84, 69.70, 68.76, 60.95, 50.82, 49.38, 38.81, 37.58, 25.53, 25.29, 24.77, 24.15.

MS (ES+): *m/z* calc. $C_{32}H_{38}O_{12}$: 614.2; found: 637.6 $[M+Na]^+$.

Appendix I- Computational data

Computational methods

All the calculations were carried out using Gaussian 16 program[3]. The geometries were optimized using ωB97X-D[4] functional with 6-31G(d)[5,6] basis set with SMD solvent model[7] to describe the DMSO environment. Intrinsic Reaction Coordination (IRC)[8] was used to confirm all transition located are connected the correct minimum. Single point energies were calculated using CAM-B3LYP-D3(BJ)[9-11] functional, 6-311++G(2d,2p) basis set[5,6] and SMD solvent model[7]. The stability of wavefunction of diradical species were checked to ensure we converged to the lowest energy wavefunction. Quasiharmonic[12] and concentration corrections to enthalpy and entropy were made using Paton's GoodVibes software[13]. The rate constants we reported are calculated via TST using Eyring's equation ($k = \frac{k_B T}{h} e^{-\frac{\Delta G^{\ddagger}}{RT}}$), where the free energies of **TS1** are used as the overall free energy barriers since it is the rate determine step.

Keywords of Spin Flip TDDFT calculation:

```
! BHANDHLYP def2-SVP def2/J RIJCOSX miniprint tightSCF CI-opt
%CPCM
SMD TRUE
SMDSOLVENT "DMSO"
END
%CONICAL
ETOL 1e-3
END
%geom
MaxStep 0.1 # maximum step length
Trust -0.1 # trust radius in internal coordinates
end
%tddft
IROOT 1
JROOT 2
nroots 5
SF True
end
```

Results

For each of the following Figures, the results of the computations by the methods described above are given. In each case the TS1_OO involves mainly OO cleavage. The charges and spin densities on relevant atoms indicate that this is a homolytic cleavage in the case of adamantyl and dimethyl substitution, but also involves substantial charge transfer for the spiro-cyclobutyl and oxetanyl cases. In all cases, the Int1 is a radical anion, with both more negative charge on the ketal O and spin on the cycloalkanone (or acetone) O. The very low barrier for TS2_CC involves CC cleavage and formation of excited phenoxide ester. The exothermic formation of the excited state of the product is notable, and the origin of the chemiluminescence.

Detailed computational investigation of Norbornyl-phenoxy-1,2-dioxetane and Homocubanyl-phenoxy-1,2-dioxetane

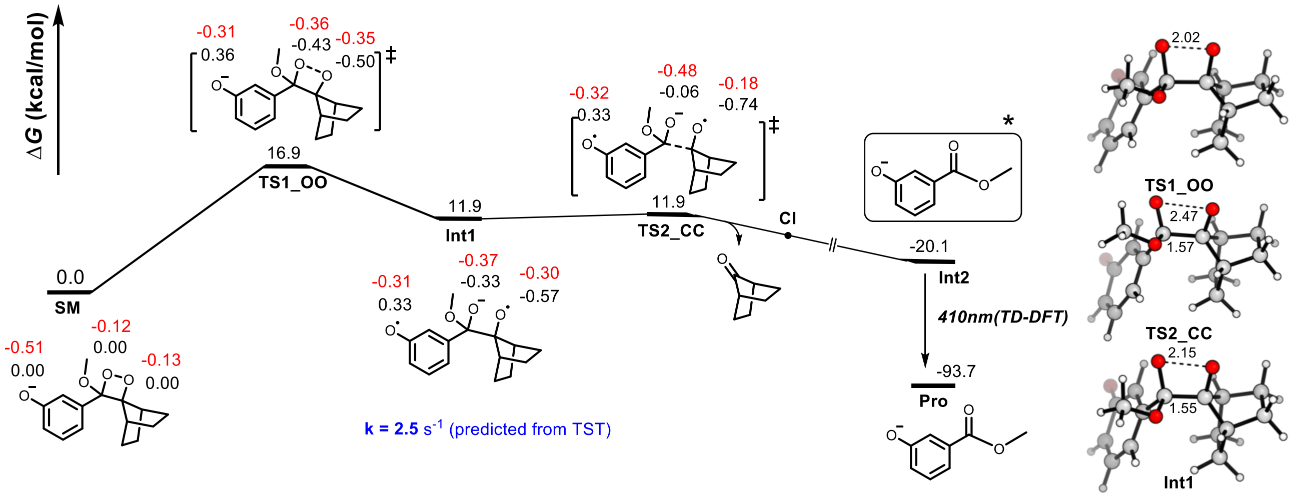

Figure S1. Complete computed free energy profile for **Diox 1**. Essential geometric features along the reaction path (in Å), Hirshfeld charges (in red), and spin populations (in black) on important atoms involved in the reaction are labeled.

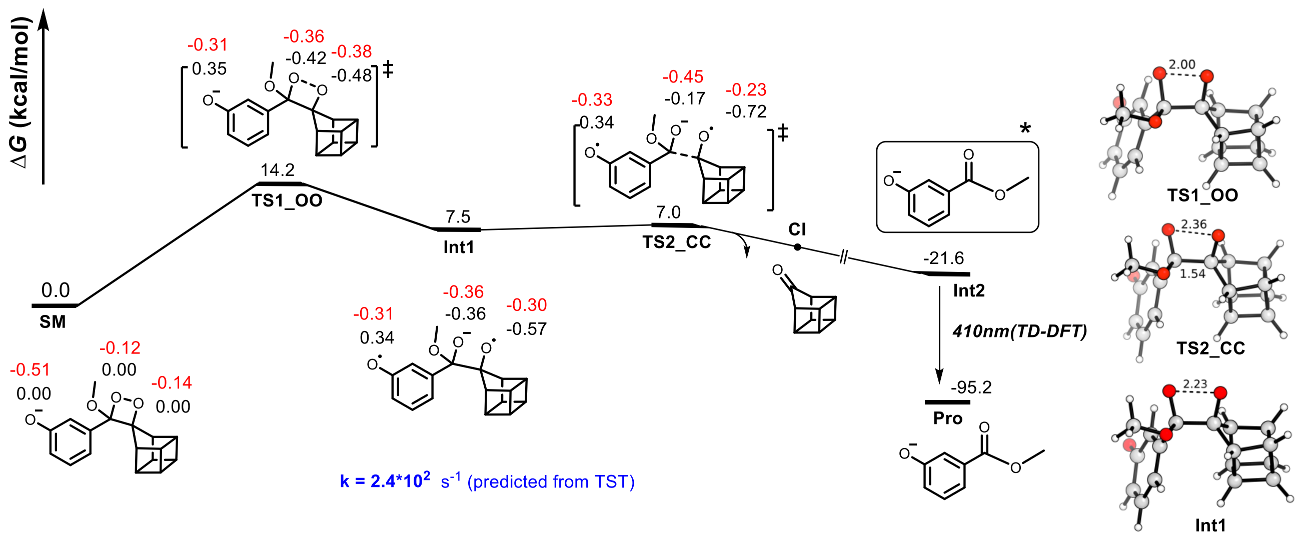

Figure S2. Complete computed free energy profile for **Diox 2**. Essential geometric features along the reaction path (in Å), Hirshfeld charges (in red), and spin populations (in black) on important atoms involved in the reaction are labeled. Red numbers below are the overall charges of the spiro-cycloalkyl fragment for the structures along the reaction pathway.

Cartesian Coordinates and Energies for Calculated Species

For all minimum structures, no imaginary frequency was observed. Energies are reported in this section directly from the output file at the optimization level of theory (ωB97X-D/6-31G(d)/SMD(DMSO)). E_SP, H and G are energies combining final single point energy with thermal corrections. (CAM-B3LYP-D3(BJ)/ 6-311++G(2d,2p)/SMD(DMSO)). All the energies here are in Hartree.

Diox1

E=-882.456909

E_SP=-882.616272

H=-882.300341

G=-882.355774

Imag. Freq. 0

Cartesian coordinates

C 0.149980 0.957849 -0.347243
O 1.591003 0.462079 -1.767912
O 0.295550 1.129484 -1.777258
O 0.469707 2.125603 0.339821
C -1.202630 0.388079 0.027871
C -1.659931 0.547606 1.346306
C -1.940761 -0.333202 -0.899885
C -2.875391 -0.047484 1.697107
H -1.080404 1.115627 2.067063
C -3.206285 -0.956940 -0.585938
H -1.567690 -0.445532 -1.914367
C -3.625764 -0.770098 0.781738
H -3.242836 0.062837 2.717215
O -3.876898 -1.607796 -1.435698
C -0.370617 3.235296 0.063539
H -1.414584 3.035070 0.329977
H 0.002466 4.060911 0.673107
H -0.314322 3.517405 -0.994058
H -4.569838 -1.222413 1.083324
C 1.388154 0.049887 -0.392145
C 1.299063 -1.467517 -0.188969

C 2.585065 0.258251 0.534638
C 2.720108 -1.887672 -0.610178
C 1.194710 -1.562868 1.344667
H 0.495234 -1.967021 -0.732399
C 3.607207 -0.716581 -0.086509
C 2.042728 -0.350615 1.838674
H 2.930272 1.288274 0.621882
H 2.994538 -2.854768 -0.177887
H 2.792581 -1.977425 -1.698370
H 0.156820 -1.475858 1.677015
H 1.582495 -2.520717 1.705296
H 4.342842 -1.042785 0.655493
H 4.154366 -0.240267 -0.906059
H 1.415682 0.374199 2.367667
H 2.853650 -0.646440 2.511437

Diox1_Int1

E=-882.429774
E_SP=-882.594532
H=-882.281344
G=-882.336805
Imag. Freq. 0

Cartesian coordinates
C 0.224048 1.070588 -0.413589
O 2.219109 0.828773 -1.377326
O 0.196713 1.475242 -1.697257
O 0.474573 2.139543 0.547710
C -1.124536 0.431883 -0.022124
C -1.642452 0.508823 1.289036
C -1.856170 -0.233701 -0.977974
C -2.852982 -0.116876 1.643687
H -1.089365 1.073333 2.032180
C -3.103038 -0.900614 -0.663120
H -1.497416 -0.270495 -2.001267
C -3.572835 -0.817687 0.707393
H -3.212800 -0.037918 2.665808
O -3.753520 -1.522233 -1.536820
C -0.254233 3.306069 0.278854
H -1.344862 3.155059 0.360426
H 0.040785 4.050723 1.026045
H -0.042928 3.694572 -0.724358
H -4.506552 -1.314255 0.954495
C 1.489826 0.183758 -0.382670
C 1.332345 -1.352394 -0.622017
C 2.327832 -0.019843 0.905766
C 2.815799 -1.748708 -0.737853
C 0.814160 -1.901834 0.720569
H 0.727939 -1.631796 -1.490284
C 3.496629 -0.865083 0.353805
C 1.444234 -0.933470 1.764836
H 2.633857 0.905807 1.396584
H 2.969239 -2.821432 -0.572378
H 3.201846 -1.497799 -1.729135
H -0.277737 -1.897420 0.778247
H 1.138198 -2.937840 0.875459
H 3.969441 -1.462385 1.142786
H 4.268062 -0.226287 -0.086384
H 0.672306 -0.355356 2.282839
H 2.023242 -1.462580 2.530882

Diox1_TS1

E=-882.412582
E_SP=-882.586178
H=-882.273969
G=-882.328908
Imag. Freq. -539.78

Cartesian coordinates
C 0.196029 1.018224 -0.403607
O 2.048885 0.620114 -1.560387
O 0.147788 1.279320 -1.772563
O 0.526303 2.153598 0.355971
C -1.164122 0.438497 -0.014897

C -1.701740 0.727416 1.246999
C -1.835470 -0.389491 -0.907820
C -2.920478 0.133275 1.590675
H -1.174430 1.376886 1.936621
C -3.097248 -1.021889 -0.595185
H -1.405706 -0.597482 -1.883360
C -3.597881 -0.707004 0.720778
H -3.346046 0.335111 2.573449
O -3.699036 -1.779361 -1.406164
C -0.198622 3.325257 0.032136
H -1.281757 3.150505 0.021066
H 0.032010 4.054870 0.812115
H 0.107118 3.729271 -0.939355
H -4.544488 -1.155923 1.018798
C 1.442204 0.098217 -0.410624
C 1.281256 -1.441118 -0.409664
C 2.432245 0.112044 0.783383
C 2.747660 -1.868749 -0.598858
C 0.882317 -1.737893 1.049368
H 0.595541 -1.841744 -1.158282
C 3.532315 -0.843269 0.274238
C 1.605068 -0.617914 1.852449
H 2.778370 1.104605 1.069228
H 2.905106 -2.904630 -0.283178
H 3.042571 -1.789529 -1.648976
H -0.199953 -1.692593 1.191561
H 1.213567 -2.738878 1.344212
H 4.050759 -1.317163 1.114003
H 4.282564 -0.308831 -0.316495
H 0.882669 0.063215 2.311981
H 2.249376 -1.010682 2.645531

Diox1_TS2

E=-882.426767
E_SP=-882.593118
H=-882.281758
G=-882.336816
Imag. Freq. -179.16

Cartesian coordinates
C 0.202618 1.093393 -0.443397
O 2.455598 1.074806 -0.914795
O 0.143047 1.493327 -1.689352
O 0.404294 2.177107 0.565299
C -1.119538 0.401541 -0.007072
C -1.602278 0.426551 1.318299
C -1.892933 -0.207923 -0.968897
C -2.813067 -0.199421 1.679827
H -1.025422 0.958795 2.067504
C -3.136365 -0.877704 -0.650340
H -1.562952 -0.185954 -2.002724
C -3.566807 -0.851403 0.736143
H -3.144612 -0.158938 2.713987
O -3.820004 -1.451968 -1.532041
C -0.351279 3.316215 0.285045
H -1.431622 3.174630 0.480325
H 0.001960 4.122966 0.939049
H -0.239984 3.619707 -0.763435
H -4.499819 -1.347295 0.987797
C 1.534037 0.270196 -0.301316
C 1.539648 -1.191348 -0.865308
C 2.177019 -0.190493 1.058037
C 3.038285 -1.535378 -0.778970
C 0.822438 -2.016713 0.221812
H 1.110475 -1.286050 -1.865933
C 3.469028 -0.882339 0.569571
C 1.221873 -1.294055 1.536565
H 2.334970 0.618604 1.773017
H 3.202490 -2.618609 -0.793840
H 3.590440 -1.098797 -1.614201
H -0.261322 -2.028041 0.086993
H 1.160893 -3.058942 0.204595
H 3.814513 -1.623081 1.300069
H 4.278001 -0.162407 0.420778
H 0.348279 -0.879411 2.045702

H 1.724160 -1.960071 2.247435

Diox1_ketone_product

E=-347.885256

E_SP=-347.923709

H=-347.756311

G=-347.791156

Imag. Freq. 0

Cartesian coordinates

C 0.756980 -1.251301 0.783197
C -0.065049 -0.000073 1.154287
C -0.065050 -0.000158 -1.154287
C 0.756943 -1.251383 -0.783108
H 1.767852 -1.196186 1.198029
H 0.283029 -2.157262 1.173354
H 1.767797 -1.196367 -1.197998
H 0.282921 -2.157359 -1.173144
C 0.755125 1.252347 0.783103
H 0.279784 2.157633 1.173136
H 1.766060 1.198811 1.197989
C 0.755159 1.252266 -0.783193
H 0.279889 2.157537 -1.173346
H 1.766113 1.198632 -1.198024
H -0.495566 -0.000344 2.155469
H -0.495568 -0.000495 -2.155469
C -1.064322 -0.000912 0.000000
O -2.272629 -0.001164 0.000001

Diox2

E=-957.321008

E_SP=-957.478014

H=-957.174896

G=-957.229849

Imag. Freq. 0

Cartesian coordinates

C 1.066560 0.683193 -0.599340
C -0.325566 1.231185 -0.270609
O 1.124043 1.601359 -1.720125
O -0.286370 1.932317 -1.538895
O -0.227395 2.113305 0.800153
C 1.242076 -0.783621 -0.968986
C 2.207967 0.783835 0.396615
H 0.552217 -1.199185 -1.703063
C 1.411073 -1.537929 0.376233
C 2.765586 -0.996315 -1.195902
H 2.371311 1.749239 0.871561
C 2.071370 -0.461848 1.312961
C 3.426740 0.074766 -0.259778
H 0.613438 -2.164986 0.773653
H 3.170815 -1.149243 -2.196413
H 1.717088 -0.366338 2.339588
H 4.279929 0.646224 -0.626357
C -1.491812 0.274096 -0.157128
C -1.762353 -0.283821 1.103157
C -2.229898 -0.088713 -1.273786
C -2.793979 -1.222475 1.197368
H -1.184574 0.011362 1.973563
C -3.315428 -1.042324 -1.220802
H -1.996200 0.350006 -2.240128
C -3.545716 -1.593636 0.092692
H -3.014506 -1.670692 2.166031
O -3.993058 -1.362656 -2.237211
C -1.343903 2.972614 0.971966
H -2.253255 2.414149 1.221310
H -1.093047 3.638421 1.800298
H -1.522147 3.573819 0.072728
H -4.345271 -2.326349 0.195782
C 3.482609 -0.975946 0.891683
H 4.325448 -1.123840 1.566893
C 2.828999 -2.037539 -0.036105
H 3.120064 -3.082260 -0.144976

Diox2_Int1

E=-957.299415

E_SP=-957.462216

H=-957.162097

G=-957.21787

Imag. Freq. 0

Cartesian coordinates

C	1.143592	0.839767	-0.447305
C	-0.273048	1.410785	-0.290914
O	1.727891	1.828059	-1.228428
O	-0.485032	1.995475	-1.479369
O	-0.203112	2.338052	0.836747
C	1.224046	-0.561746	-1.090859
C	2.017654	0.489531	0.768424
H	0.676416	-0.701932	-2.024890
C	1.034256	-1.628682	0.017797
C	2.731884	-0.934517	-1.089449
H	2.173727	1.281500	1.501217
C	1.578803	-0.902851	1.299948
C	3.278598	-0.214685	0.193317
H	0.119182	-2.218871	0.096637
H	3.328259	-0.909831	-2.002978
H	1.032044	-1.008046	2.238557
H	4.243441	0.293198	0.144522
C	-1.360827	0.378095	0.062263
C	-1.583790	-0.025566	1.397990
C	-2.108344	-0.199329	-0.935066
C	-2.520251	-1.025675	1.719126
H	-1.016823	0.458858	2.186058
C	-3.085280	-1.233039	-0.657768
H	-1.964372	0.116728	-1.963385
C	-3.255022	-1.630931	0.728266
H	-2.657932	-1.313563	2.757722
O	-3.757771	-1.765361	-1.572299
C	-1.255521	3.262148	0.847758
H	-2.234637	2.788112	1.038723
H	-1.059982	3.972220	1.658739
H	-1.323574	3.805871	-0.102377
H	-3.982036	-2.407667	0.946387
C	2.993421	-1.501476	1.027264
H	3.655035	-1.922434	1.786181
C	2.454808	-2.216016	-0.243220
H	2.665573	-3.238812	-0.559752

Diox2_TS1

E=-957.279069

E_SP=-957.45186

H=-957.152464

G=-957.207201

Imag. Freq. -531.87

Cartesian coordinates

C	1.090895	0.832831	-0.634060
C	-0.332072	1.340501	-0.324043
O	1.454643	1.839379	-1.544669
O	-0.542009	1.979874	-1.551689
O	-0.233034	2.248827	0.740595
C	1.244408	-0.589341	-1.179130
C	2.102392	0.700407	0.512597
H	0.644428	-0.855812	-2.048691
C	1.208202	-1.541810	0.041289
C	2.774158	-0.855666	-1.254841
H	2.242358	1.574265	1.145452
C	1.787538	-0.657106	1.200576
C	3.359255	0.020362	-0.093811
H	0.340214	-2.170468	0.241002
H	3.296295	-0.882960	-2.211478
H	1.308693	-0.686917	2.179291
H	4.276976	0.584129	-0.263609
C	-1.415234	0.291149	-0.097049
C	-1.731909	-0.072393	1.219790
C	-2.027517	-0.326515	-1.180069
C	-2.668587	-1.093692	1.408800

H -1.250227 0.418250 2.058360
C -3.006211 -1.379762 -1.031080
H -1.765129 -0.031999 -2.192403
C -3.284025 -1.729238 0.341334
H -2.918185 -1.398878 2.424793
O -3.560697 -1.939941 -2.017222
C -1.322126 3.145316 0.861096
H -2.279486 2.617301 0.954114
H -1.146546 3.722231 1.771976
H -1.371979 3.830350 0.007115
H -4.009089 -2.522213 0.520150
C 3.219262 -1.189222 0.880123
H 3.958503 -1.487145 1.623663
C 2.645070 -2.060202 -0.271169
H 2.905272 -3.092524 -0.504881

Diox2_TS2

E=-957.299275
E_SP=-957.462497
H=-957.163719
G=-957.218671
Imag. Freq. -165.59

Cartesian coordinates
C 1.143650 0.936399 -0.567094
C -0.316552 1.425347 -0.483534
O 1.764673 1.973782 -1.238320
O -0.557186 1.957858 -1.675656
O -0.322218 2.383526 0.634662
C 1.332565 -0.423376 -1.274164
C 1.979145 0.565442 0.674721
H 0.837662 -0.537811 -2.240115
C 1.141099 -1.554845 -0.229878
C 2.854685 -0.723244 -1.212302
H 2.057242 1.324782 1.452975
C 1.583785 -0.873345 1.111429
C 3.300005 -0.046789 0.131020
H 0.254263 -2.191157 -0.228510
H 3.493655 -0.621651 -2.090796
H 0.998968 -1.051932 2.014881
H 4.238371 0.509455 0.158581
C -1.333871 0.326186 -0.101335
C -1.542516 -0.061288 1.240828
C -2.041631 -0.314596 -1.089245
C -2.420372 -1.109038 1.578304
H -1.012748 0.474019 2.021976
C -2.956134 -1.399346 -0.796668
H -1.911068 -0.004712 -2.121394
C -3.110487 -1.778246 0.596767
H -2.548569 -1.382329 2.622155
O -3.590679 -1.989591 -1.703108
C -1.430628 3.236146 0.605291
H -2.373982 2.716852 0.855100
H -1.268868 4.018634 1.355593
H -1.551952 3.697118 -0.382843
H -3.791327 -2.592347 0.827620
C 3.039242 -1.387172 0.883466
H 3.681534 -1.815284 1.654568
C 2.600743 -2.059054 -0.446890
H 2.877745 -3.052514 -0.802649

Diox2_ketone_product

E=-422.750374
E_SP=-422.785172
H=-422.630623
G=-422.664639
Imag. Freq. 0

Cartesian coordinates
C 1.231647 -0.561478 -1.221738
C 2.070896 0.709233 0.543930
H 0.643719 -0.788341 -2.109167
C 1.145611 -1.515675 0.003377
C 2.757380 -0.864401 -1.231329

H 2.208051 1.580144 1.182153
C 1.713523 -0.655671 1.198334
C 3.325294 -0.004411 -0.036337
H 0.256781 -2.123026 0.173230
H 3.313229 -0.887770 -2.168444
H 1.200595 -0.693828 2.159152
H 4.257227 0.541425 -0.182213
C 3.135917 -1.222528 0.915106
H 3.847475 -1.538939 1.677392
C 2.576299 -2.070039 -0.262441
H 2.812189 -3.106731 -0.501435
C 1.118267 0.837431 -0.635211
O 0.460724 1.782633 -1.002980

hydroxybenzoate

E=-534.684923
E_SP=-534.802009
H=-534.655292
G=-534.697792
Imag. Freq. 0

Cartesian coordinates
C -1.480500 -0.424469 0.000032
O -1.815389 -1.593454 0.000101
O -2.377678 0.575851 -0.000056
C -0.071484 0.063296 0.000015
C 0.224514 1.436348 0.000065
C 0.939542 -0.892884 -0.000030
C 1.570928 1.809056 0.000023
H -0.564028 2.179525 0.000148
C 2.341272 -0.553769 -0.000025
H 0.671346 -1.946467 -0.000054
C 2.590419 0.868228 -0.000026
H 1.826324 2.868236 0.000049
O 3.259952 -1.420225 -0.000026
C -3.748815 0.180114 -0.000062
H -3.985300 -0.406825 0.891571
H -4.323757 1.106702 -0.000096
H -3.985267 -0.406864 -0.891677
H 3.630349 1.192802 -0.000045

Appendix II- Supplementary Figures

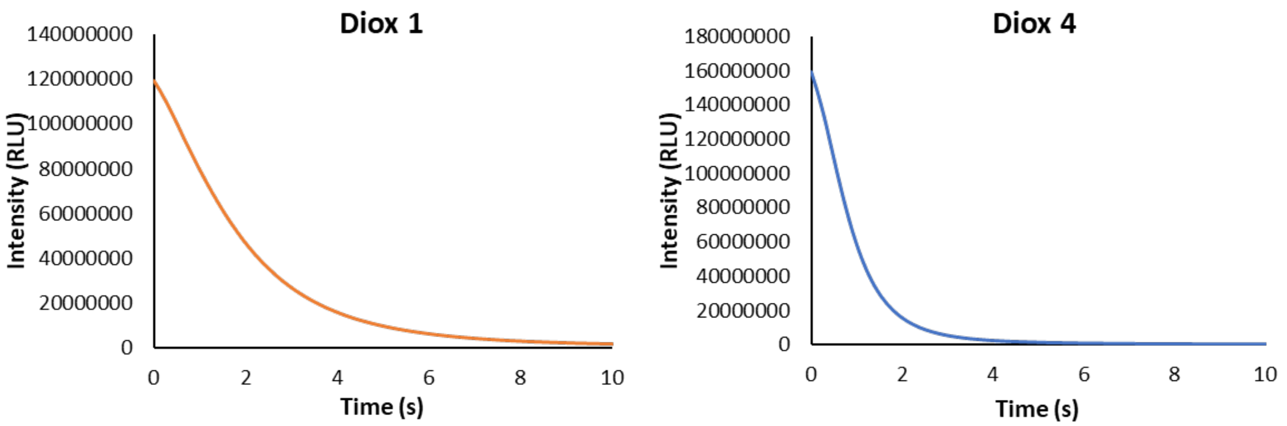

Figure **S3**: Chemiluminescent kinetic profiles during the first 5 seconds of **Diox 1** and **Diox 4** [10 nM], measured in DMSO, with TBAF [10 mM]. The detailed assay procedure is mentioned in the experimental protocols section.

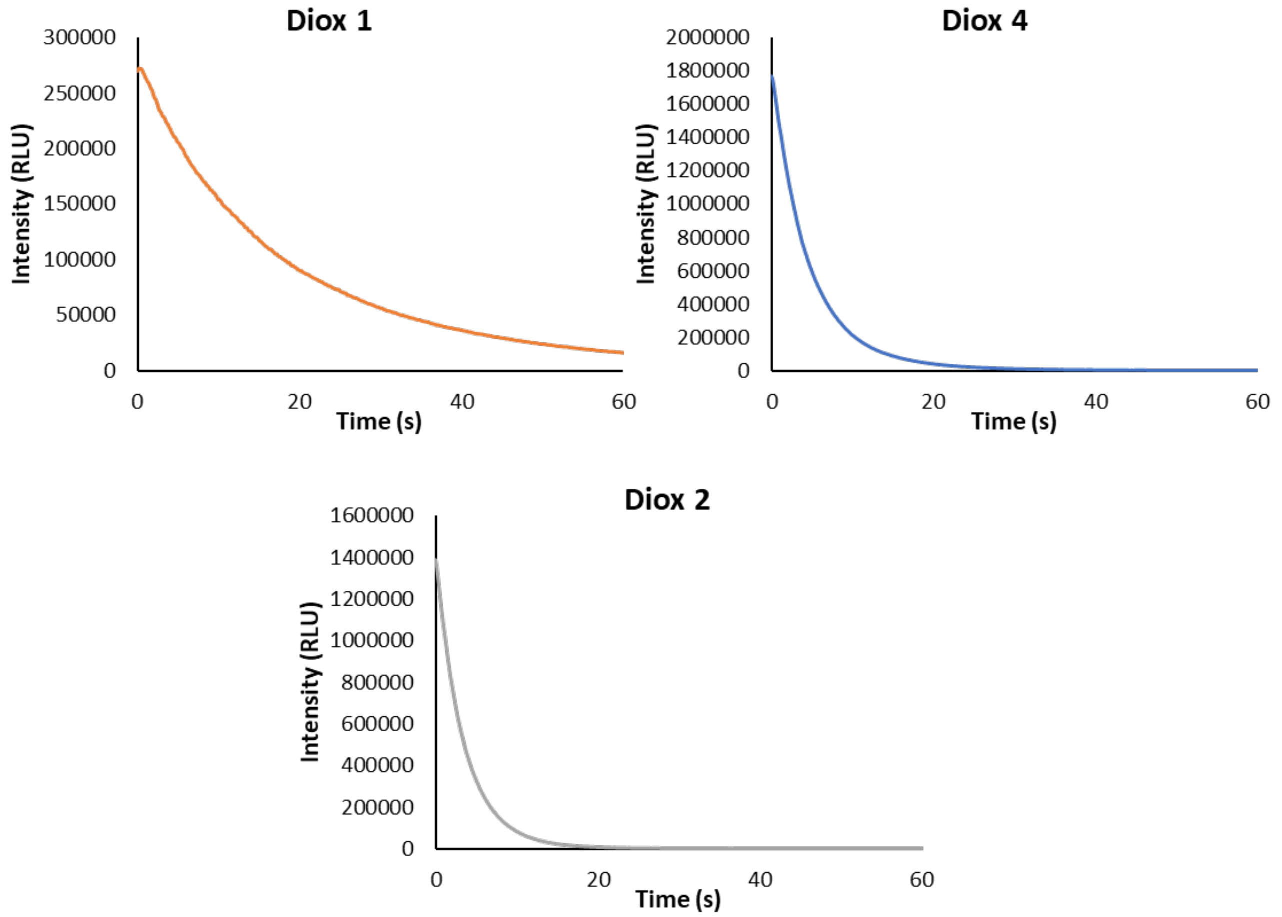

Figure **S4**: Full chemiluminescent kinetic profiles of **Diox 1**, **Diox 2** and **Diox 4** [10 nM] measured in Acetone, with TBAF [10 mM]. The detailed assay procedure is mentioned in the experimental protocols section.

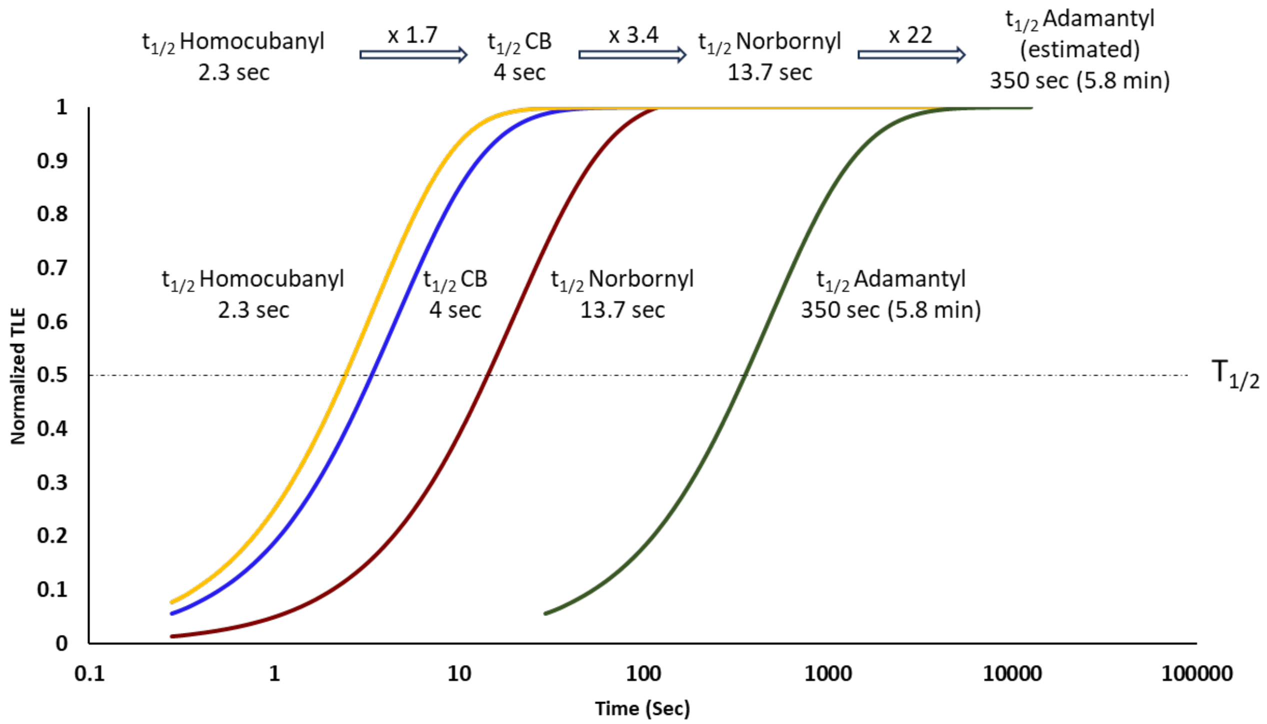

Figure **S5**: Normalized chemiluminescent total light emission profiles of Norbornyl (**Diox 1**) [10nM], Homocubanyl (**Diox 2**) [10nM], and Adamantyl (**Diox 3**) [10nM], and Cyclobutyl (**Diox 4**) [10nM] with TBAF [1mM] in Acetone at 27C.

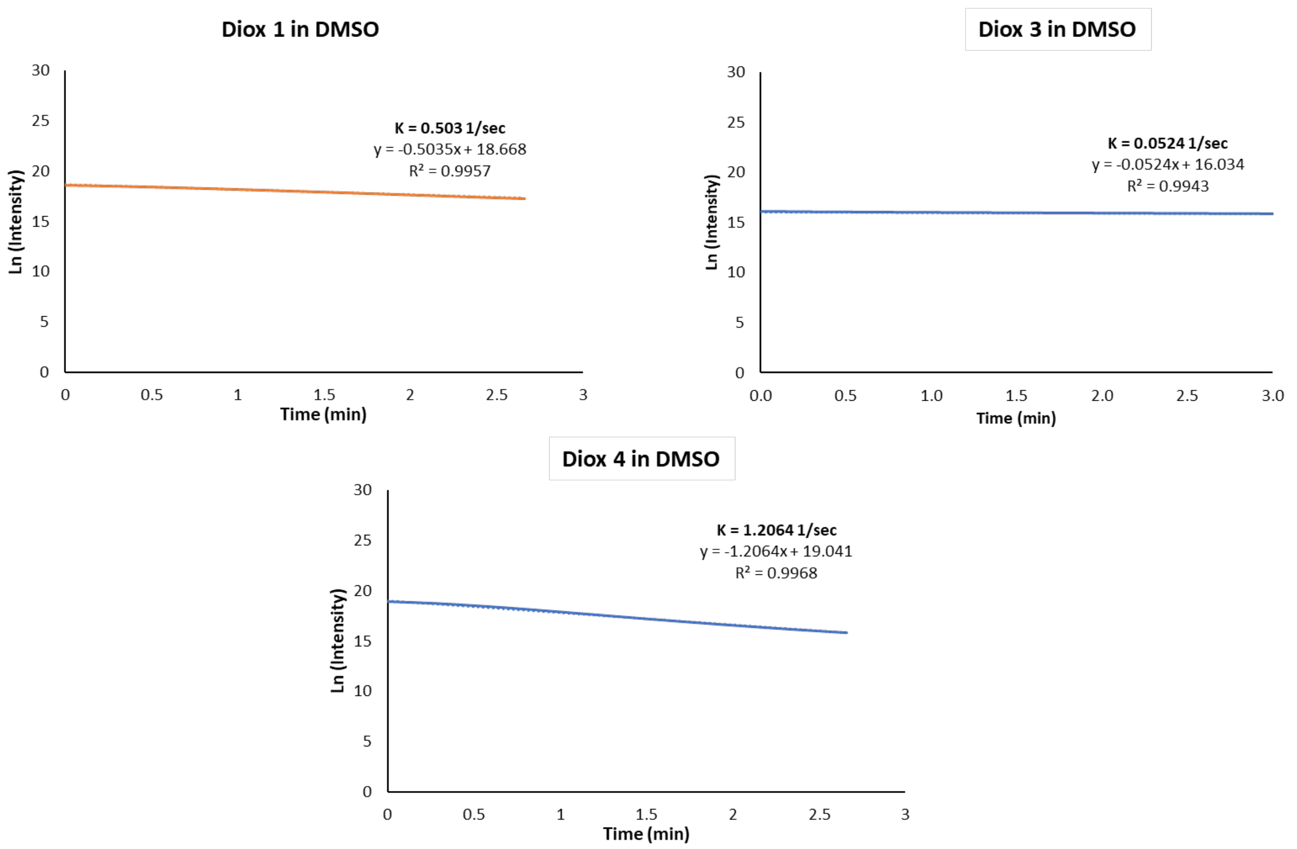

Figure **S6**: Ln of intensity vs time of Diox 1, Diox 3 and Diox 4 [10 nM] with TBAF [10 mM], in DMSO, 10% ACN. The detailed assay procedure is mentioned in the experimental protocols section.

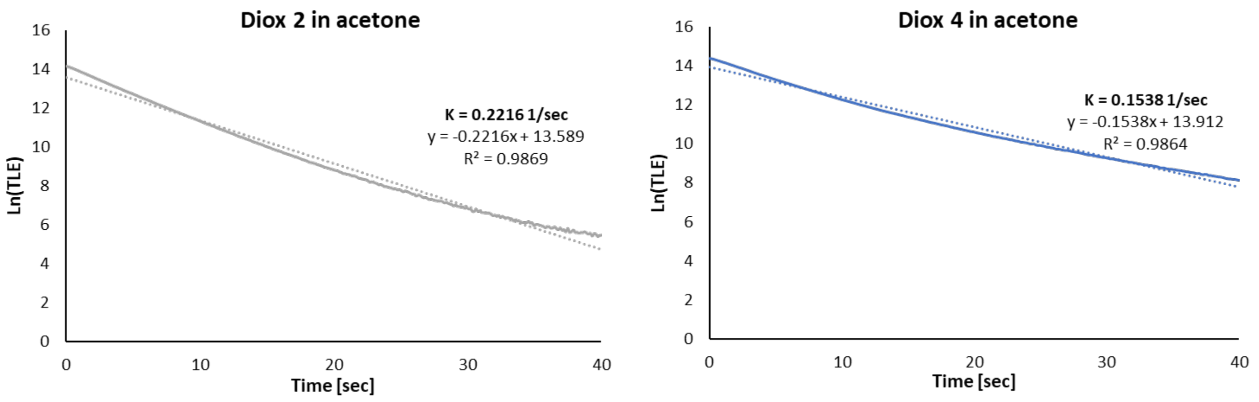

Figure **S7**: Ln of intensity vs time of Diox 2 and Diox 4 [10 nM] with TBAF [10 mM], in ACN. The detailed assay procedure is mentioned in the experimental protocols section.

Probe	K (1/sec)<br>Rate constant in DMSO	K (1/sec)<br>Rate constant in acetone
Diox 1	0.5035	-
Diox 2	-	0.2216
Diox 3	0.0524	-
Diox 4	1.2064	0.1538

Figure **S8**: Rate constants of Diox 1- Diox 4 [10 nM] with TBAF [10 mM], in DMSO/ACN.

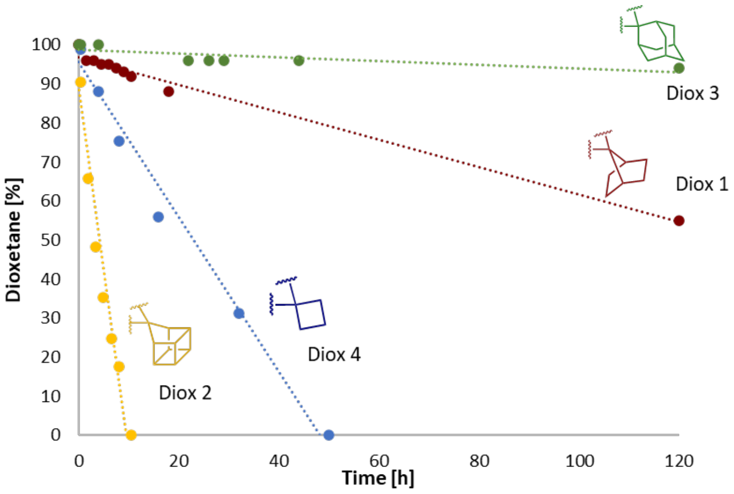

Compound	$t_{1/2}$<br>Stability at RT
**Diox 1**	**110 h**
**Diox 2**	**3 h**
**Diox 3**	**110 h**
**Diox 4**	**19.6 h**

Figure **S9**: Stability $t_{1/2}$ of **Diox 1-4** [500 μM] measured in PBS [100 mM], pH 7.4, 10% ACN at room temperature; the ratio of product distribution was determined using RP-HPLC (90-100% ACN in water with 0.1% TFA). The detailed assay procedure is mentioned in the experimental protocols section. Stability at RT is represented in hours.

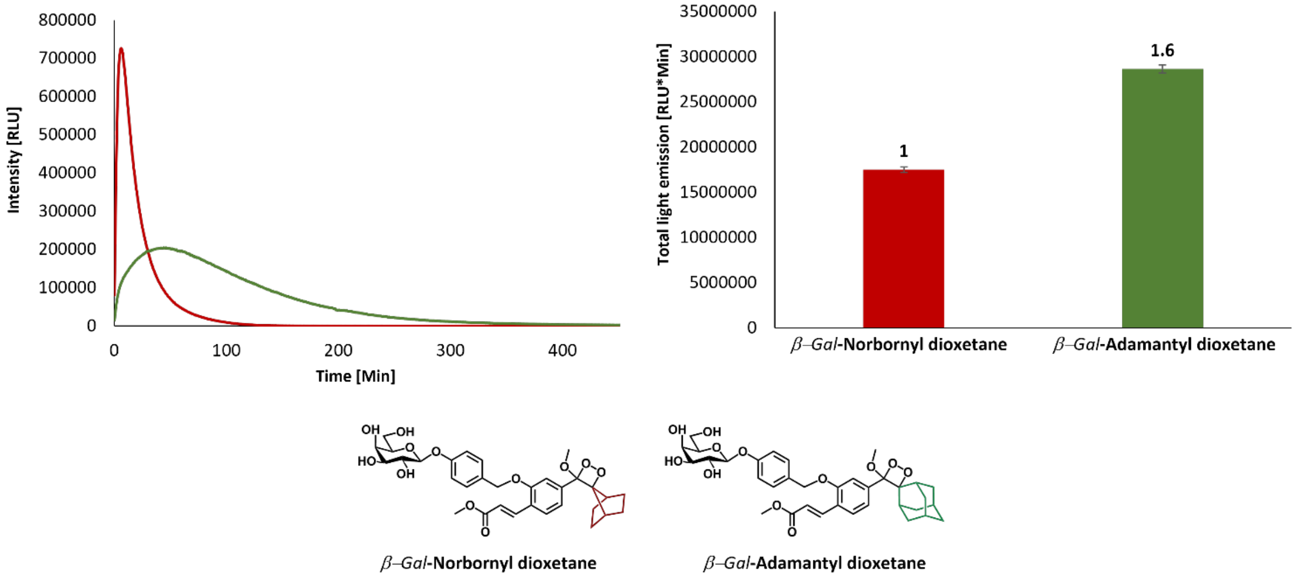

Figure **S10**. Chemiluminescence kinetic profiles (left) and Total light emission (right) of MA β-gal-Norbornyl dioxetane and MA β-gal-Adamantyl dioxetane [10uM] in PBS (7.4 pH), 1% ACN, 27°C. with and without β-galactosidase [2 U/mL].

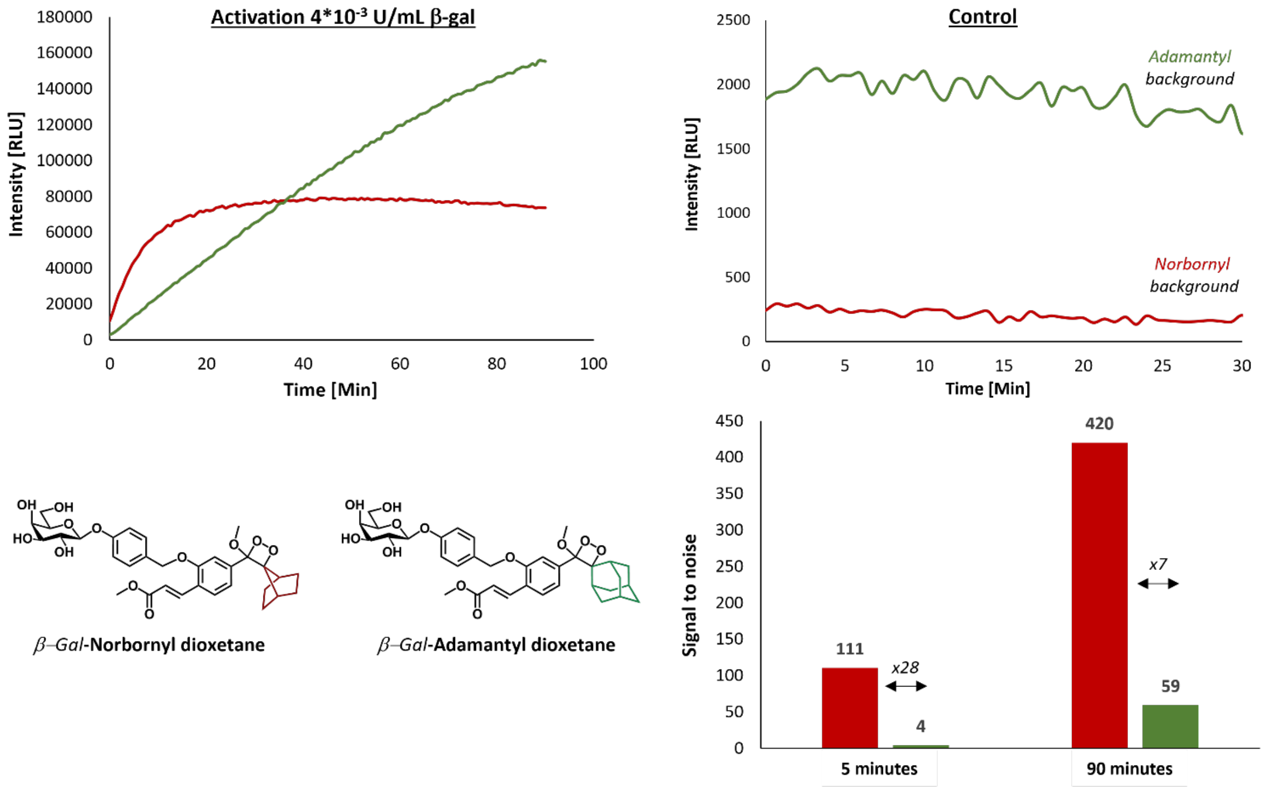

Figure **S11**. S/N ratio over time (left), Background signal (top right), and signal-to-noise ratios (bottom right) of MA β-gal-Norbornyl dioxetane and MA β -gal-Adamantyl dioxetane [10uM] in PBS (7.4 pH), 1% ACN, 27°C. with and without β-galactosidase [4E-3 U/ml].

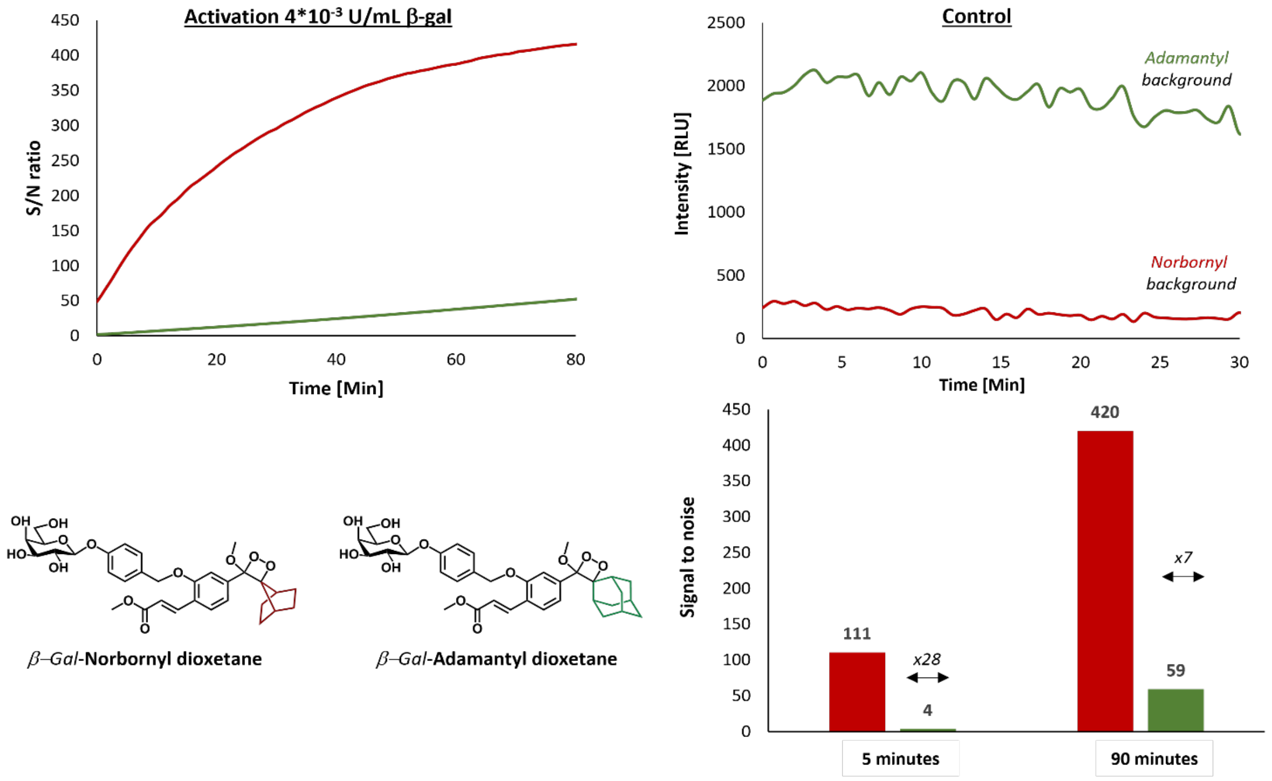

Figure **S12.** S/N ratio over time (left), Background signal (top right), and Signal-to-noise ratios (bottom right) of MA β-gal-Norbornyl dioxetane and MA β-gal-Adamantyl dioxetane [10uM] in PBS (7.4 pH), 1% ACN, 27°C. ACN, with and without β-galactosidase [4E-3 U/ml].

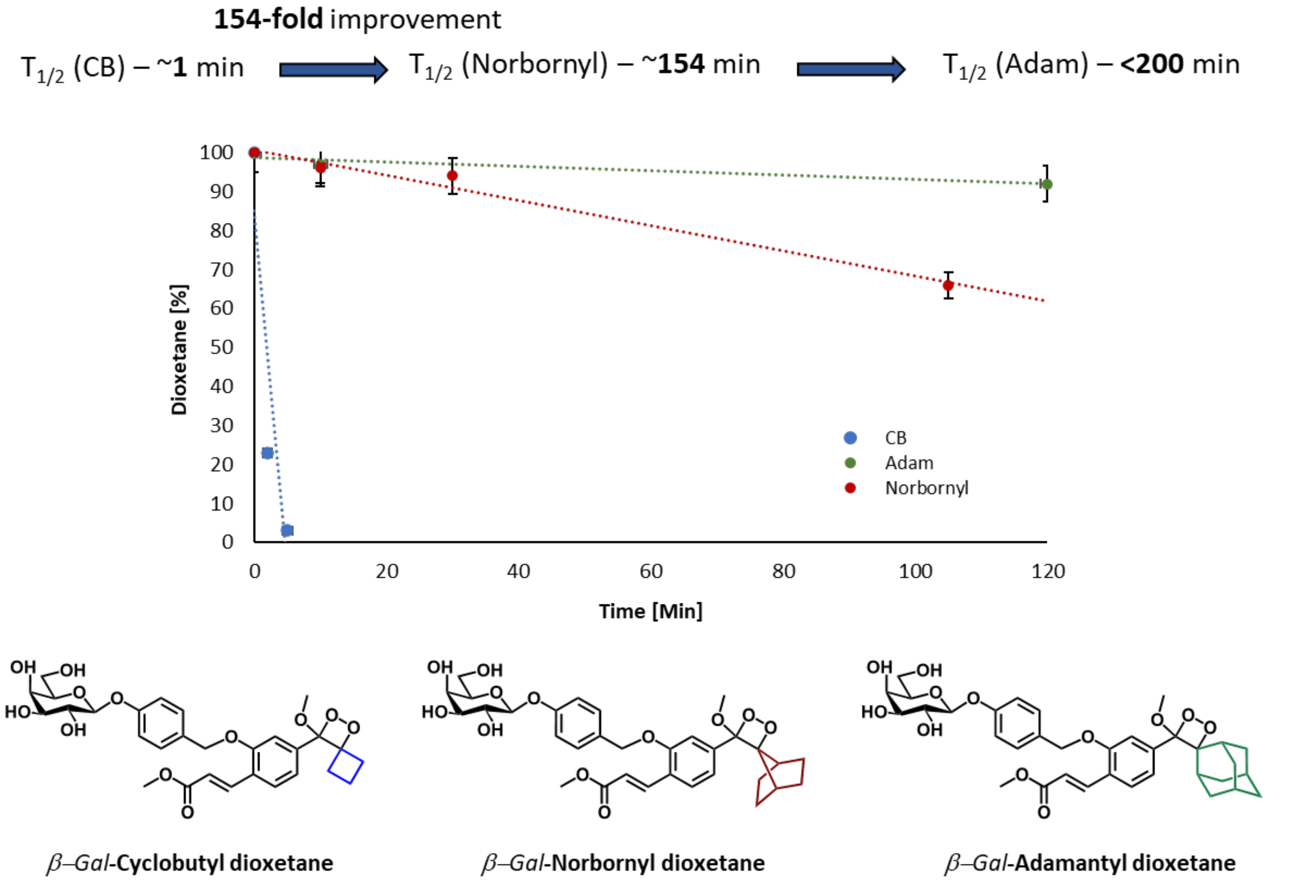

Figure **S13**. Stability assay in L.B bacteria media of MA β-gal Cyclobutyl, Norbornyl, and Adamantyl [1mM] in 95% L.B media, 5%ACN, monitored by RP-HPLC (50-100% ACN in H_2O 0.1%TFA). L.B Broth composition: 25% NaCl, 25% Yeast Extract, 50% Peptone (amino acids, vitamins, carbohydrates, and proteins).

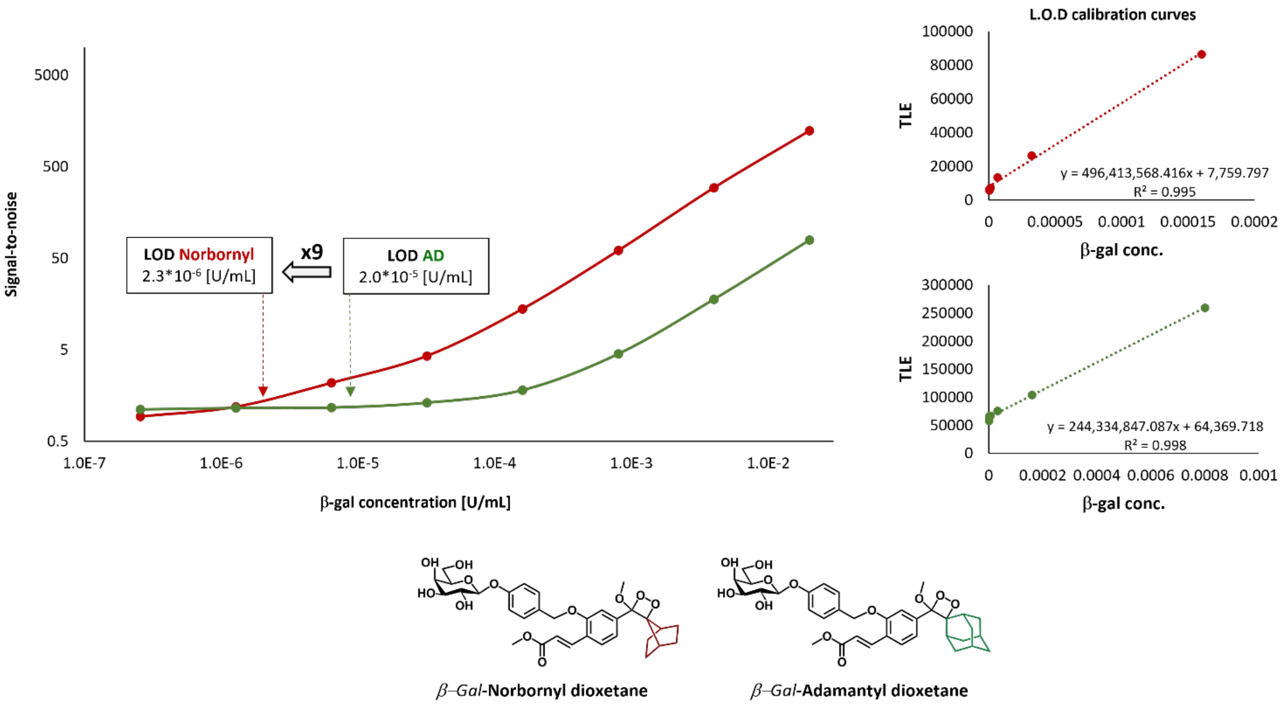

Figure **S14**. Signal-to-noise after 30 min (left) and L.O.D calibration curves(right) of MA β-gal-Norbornyl dioxetane and MA β-gal-Adamantyl dioxetane [10uM] in PBS (7.4 pH), 1% ACN, 27°C. with and without β-galactosidase [2E-2 – 2.56E-7 U/mL]. *The limit-of-detection was determined as 3*(S.D. of the blank) divided by the slope of the linear calibration curve (L.O.D = 3σ/k).

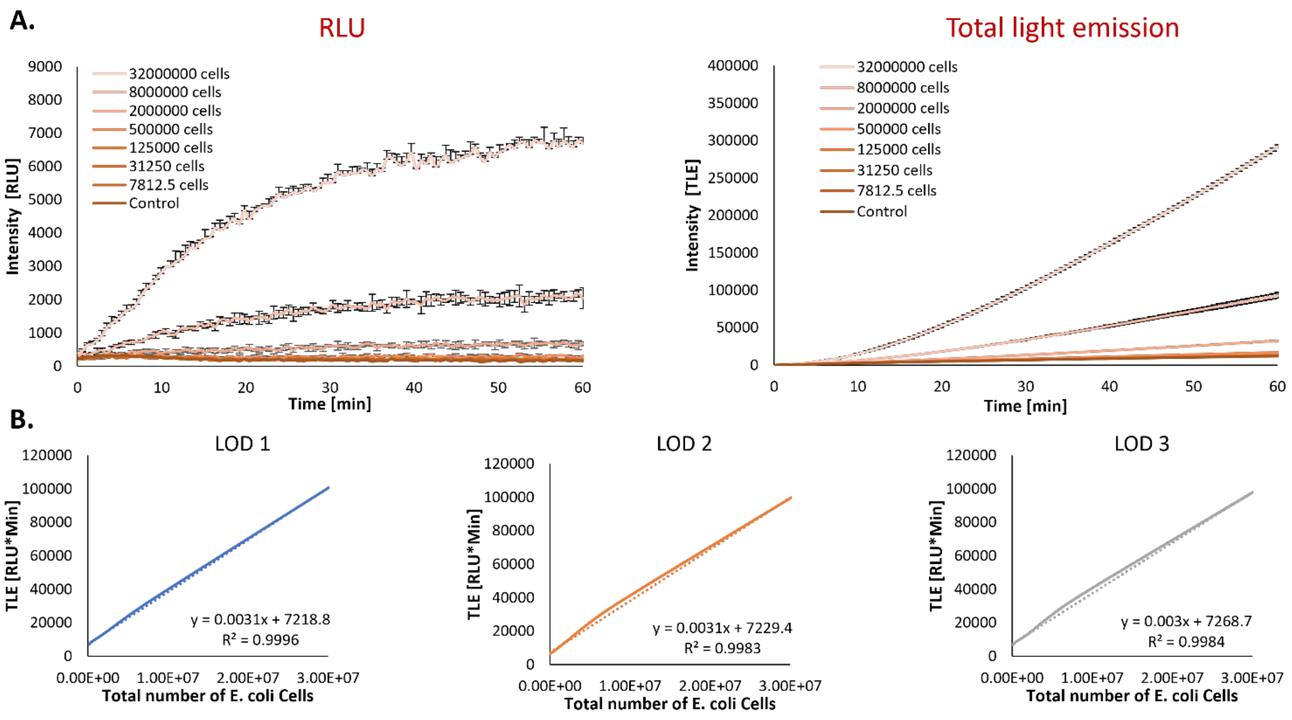

Figure S15. A. Chemiluminescence kinetic profile (left) and total light emission (right) of MA β-gal-Norbornyl [10 uM] in PBS (7.4 pH), 0.1% ACN, 37°C, with and without various concentrations of E. coli ATCC 25922 [1.95×10^3 to 3.20×10^7 cells). **B.** Determination of the limit of detection values: Total light emission vs. various concentrations of E. coli ATCC 25922 [1.95×10^3 to 3.20×10^7 cells).

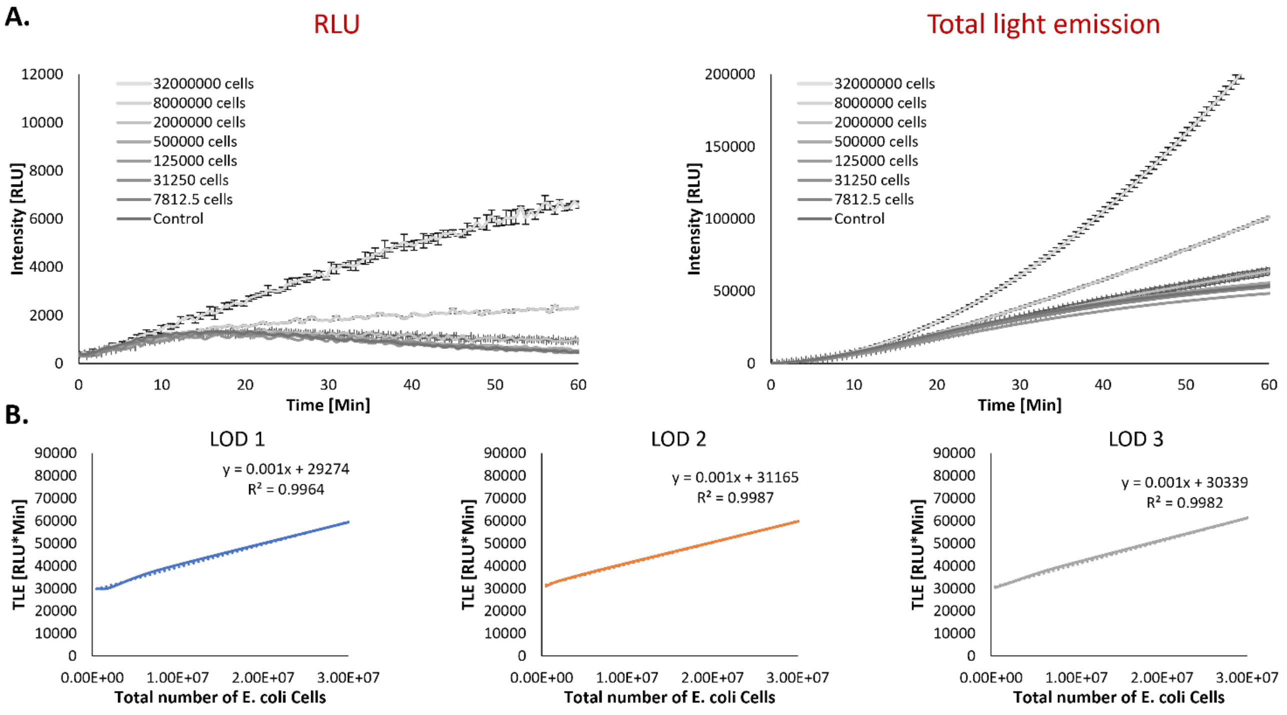

Figure S16. A. Chemiluminescence kinetic profile (left) and total light emission (right) of MA β-gal-Adamantyl [10 uM] in PBS (7.4 pH), 0.1% ACN, 37°C, with and without various concentrations of E. coli ATCC 25922 [1.95×10^3 to 3.20×10^7 cells). **B.** Determination of the limit of detection values: Total light emission vs. various concentrations of E. coli ATCC 25922 [1.95×10^3 to 3.20×10^7 cells).

Appendix III-NMR and MS Spectra

Norbornyl enolether (Compound 1a)

^{1}H-NMR

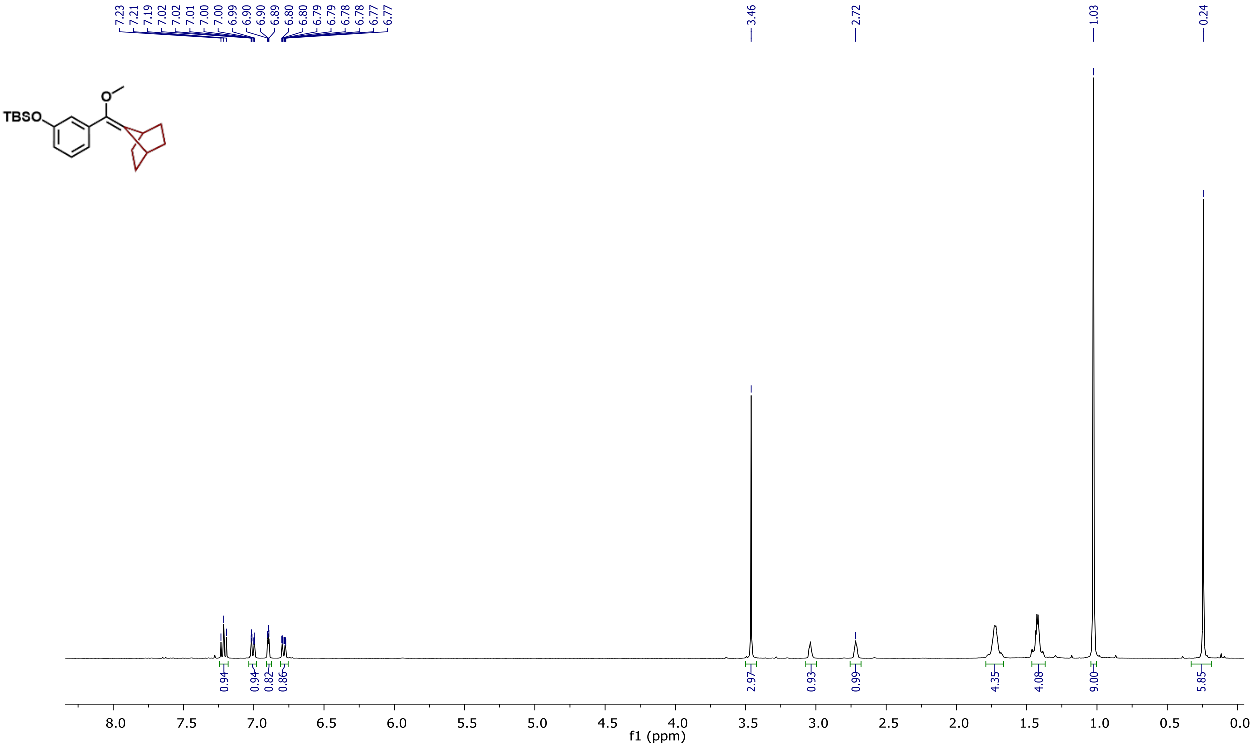

^{13}C-NMR

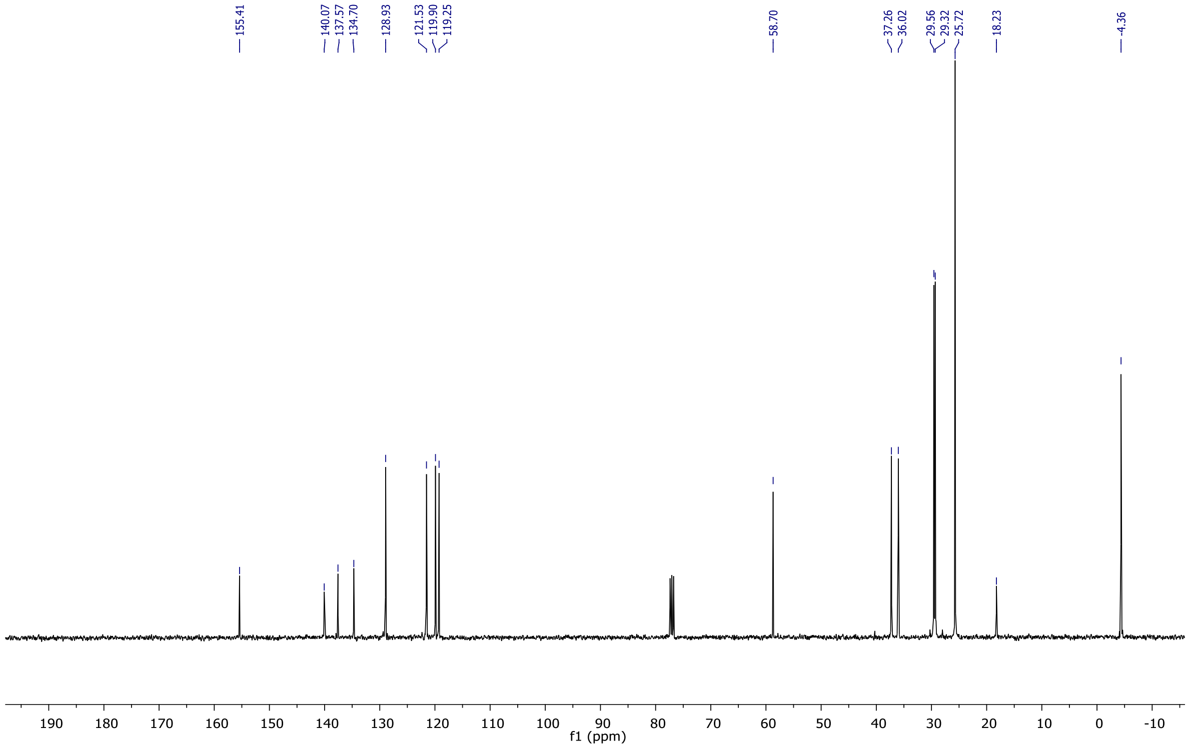

Norbornyl dioxetane (Diox 1)

^{1}H-NMR

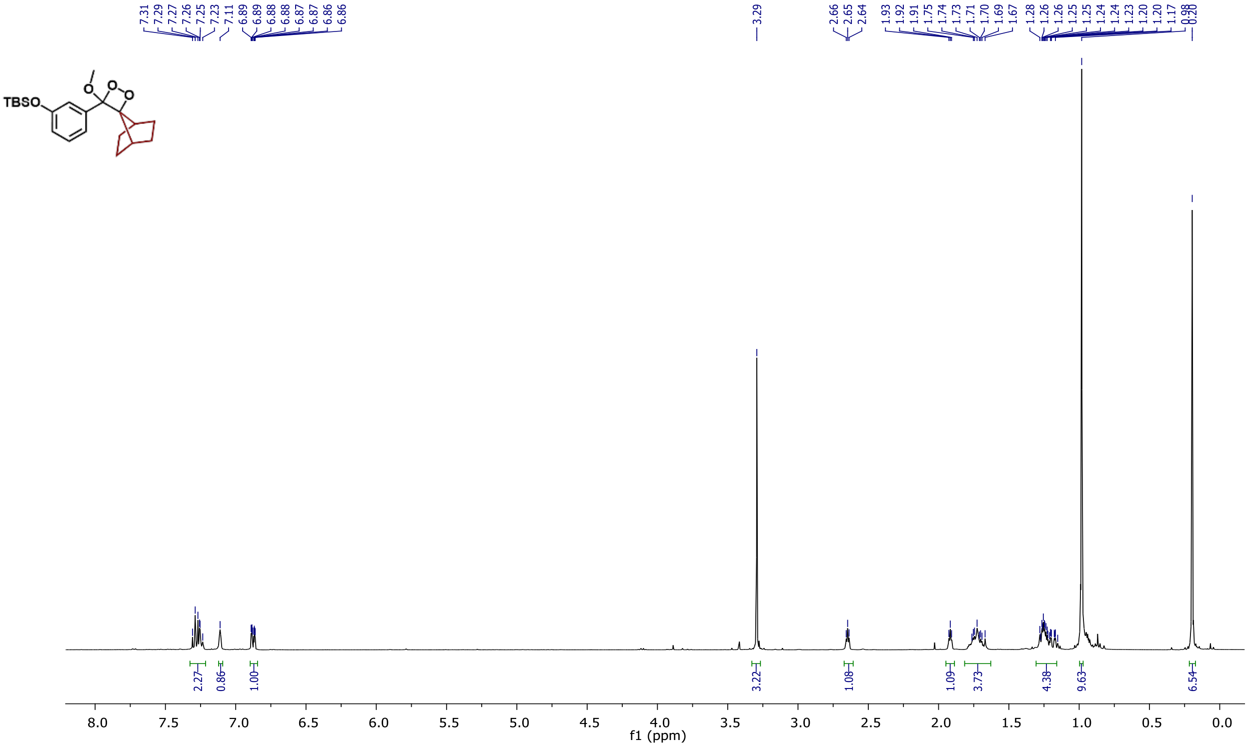

^{13}C-NMR

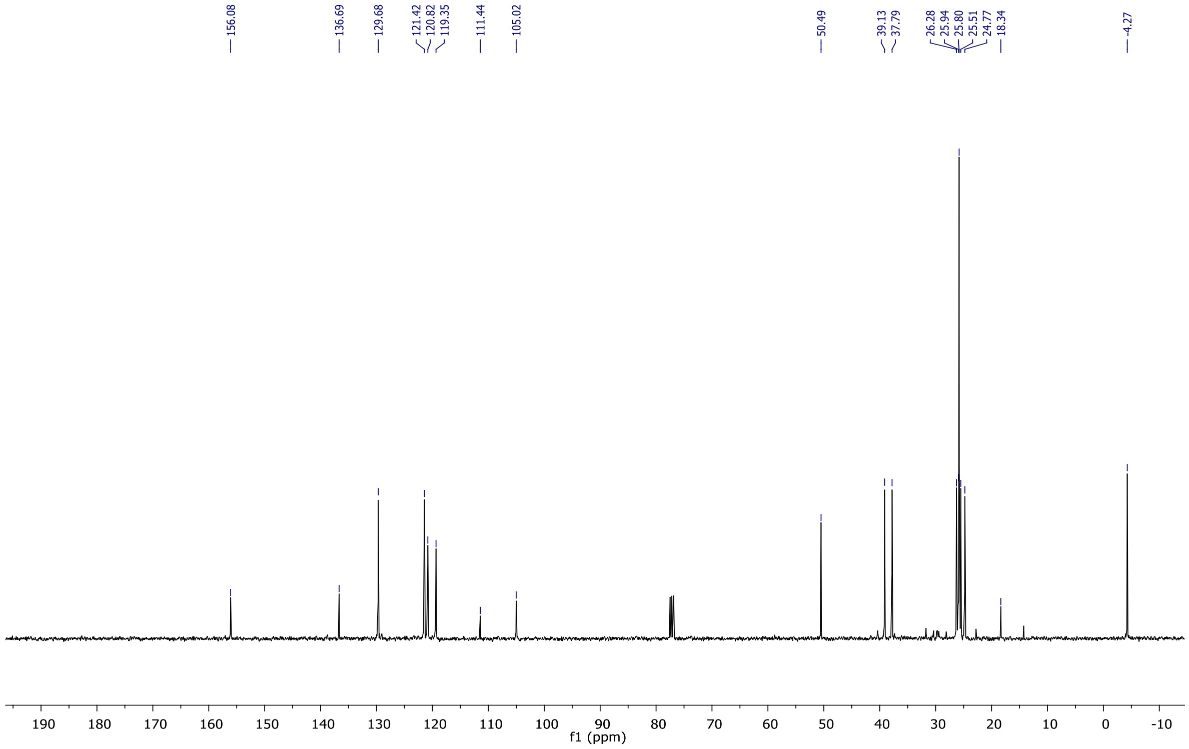

Homocubanyl enolether (Compound 2a)

^{1}H-NMR

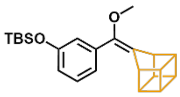

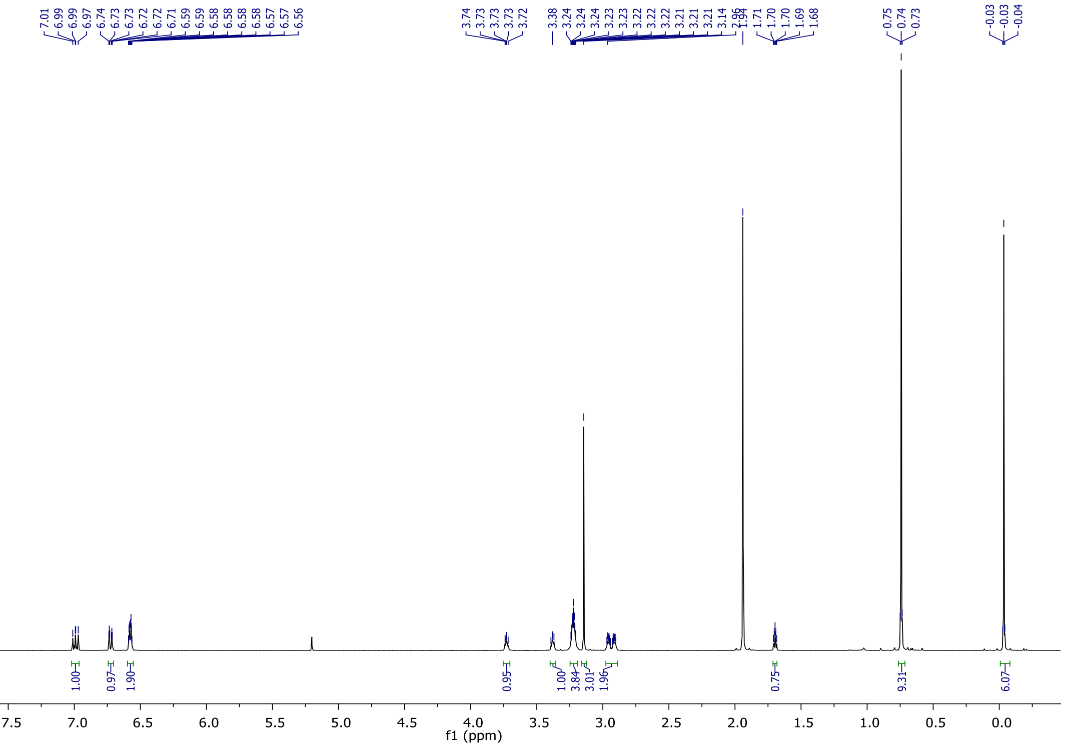

^{13}C-NMR

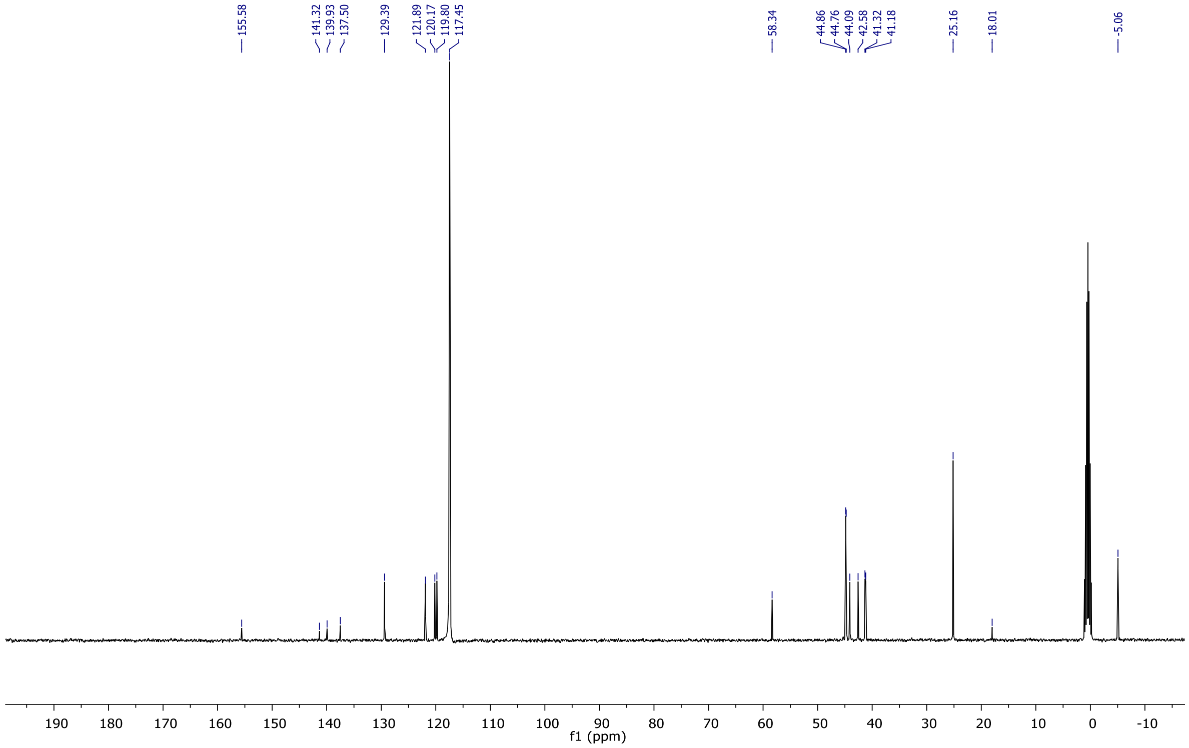

Homocubanyl dioxetane (Diox 2)

^{1}H-NMR

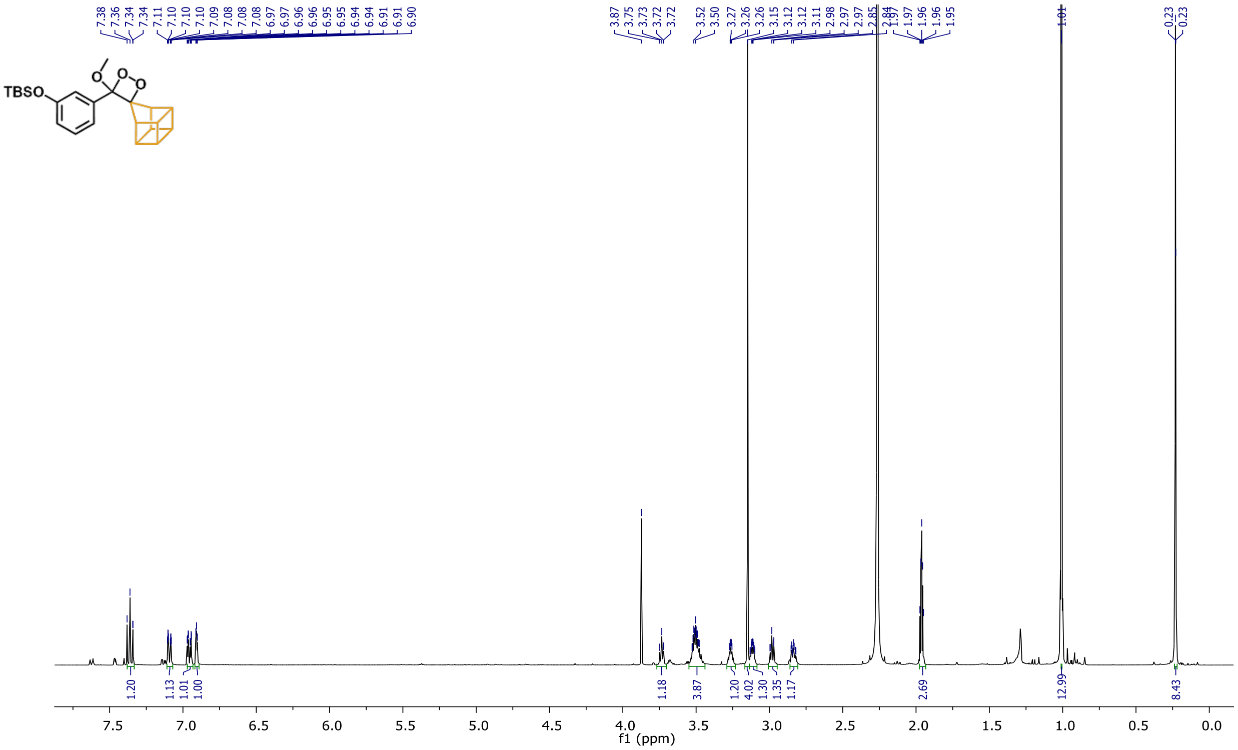

^{13}C-NMR

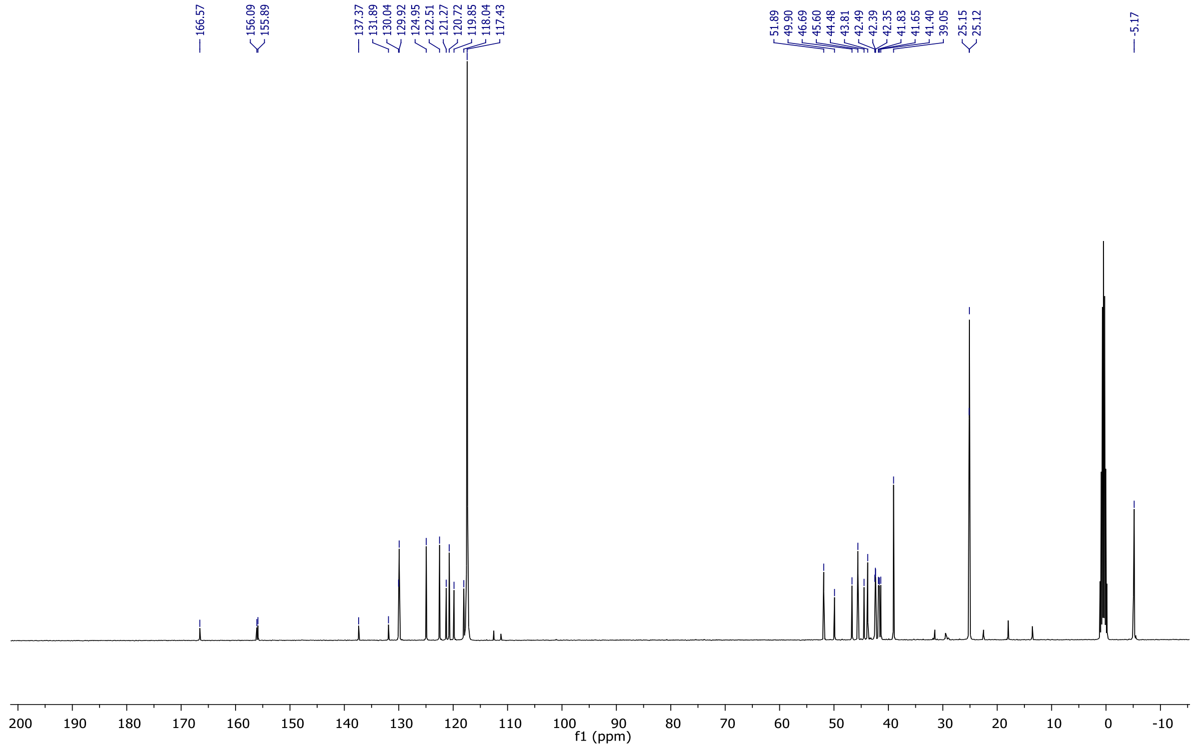

Probe MA-β-gal-norbornyl

^{1}H-NMR

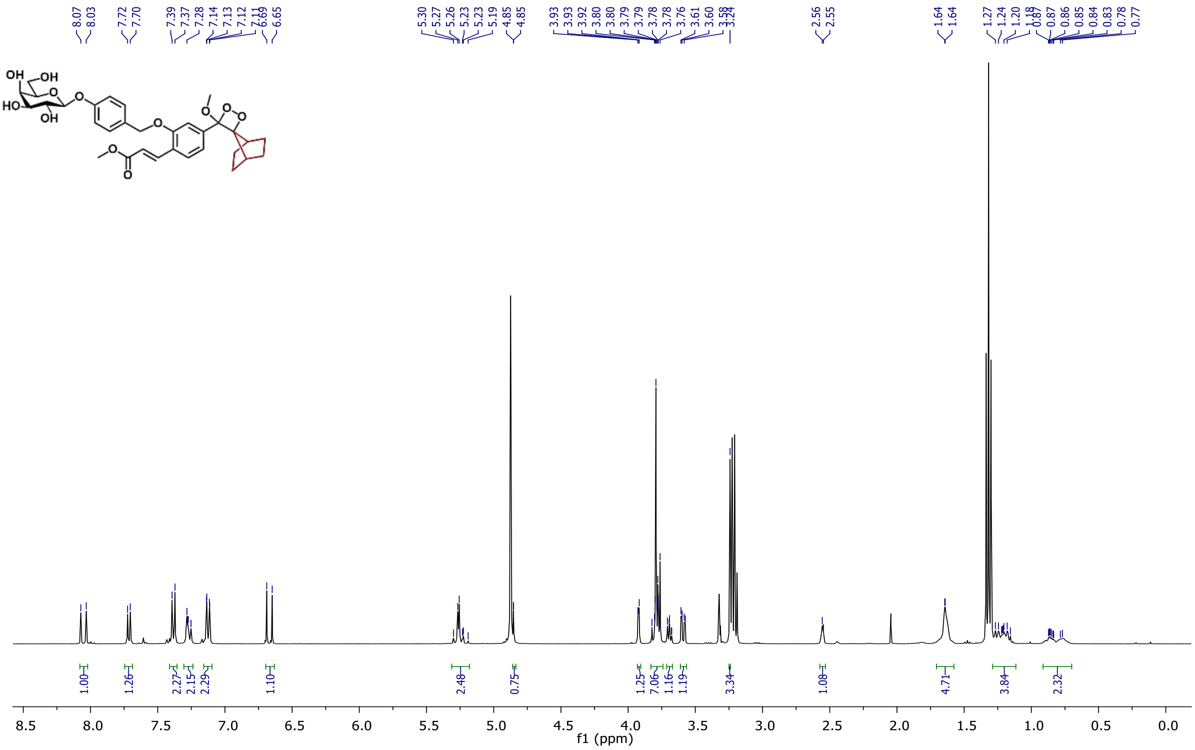

^{13}C-NMR

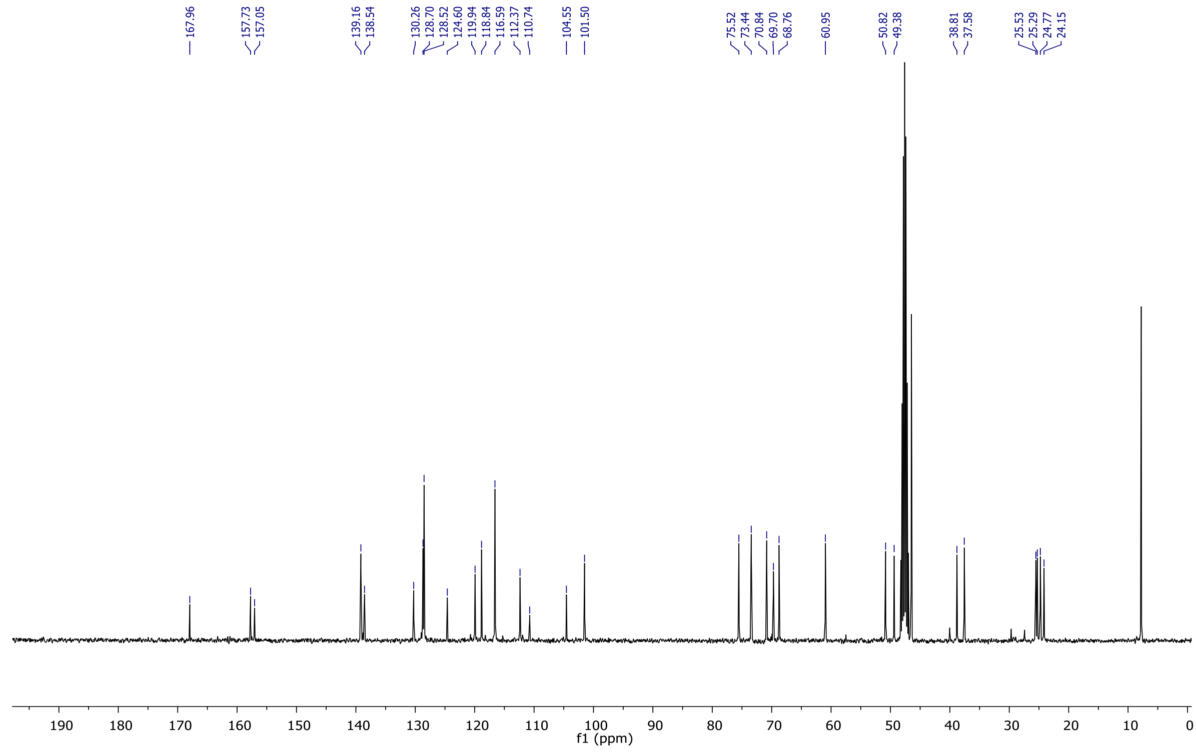

MS

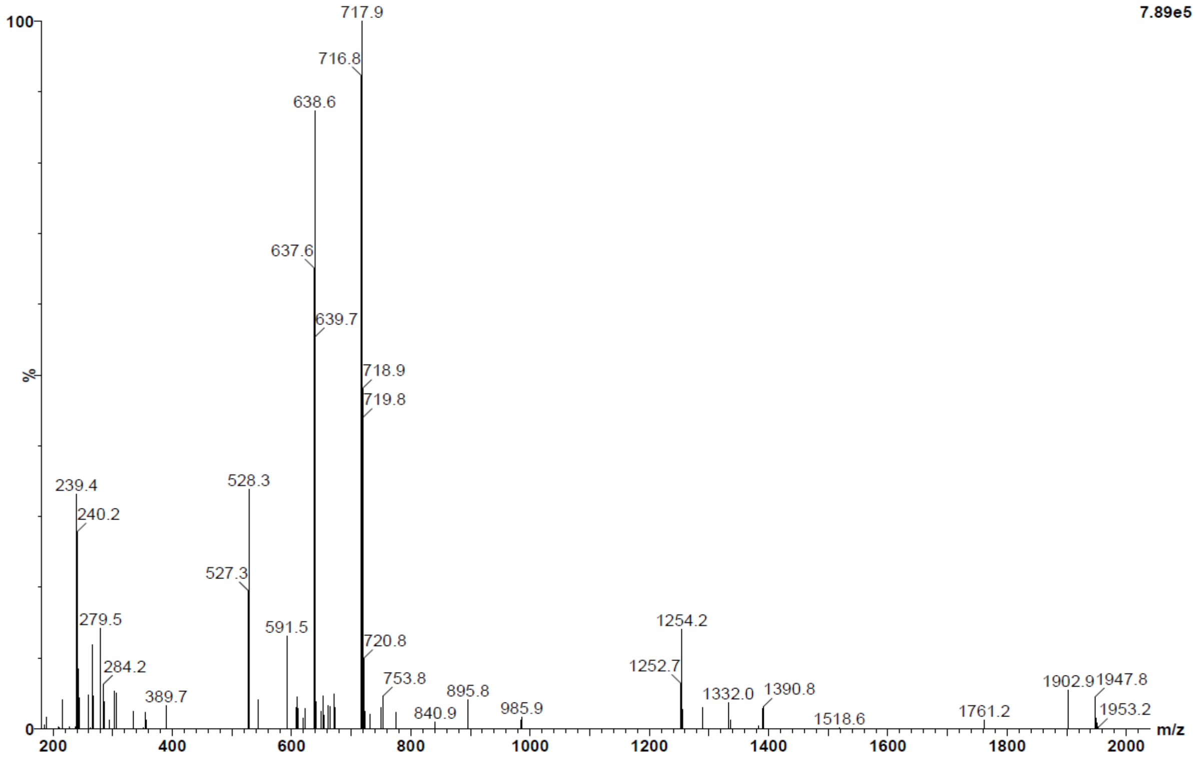

7.89e5
100
%
0
717.9
716.8
638.6
637.6
639.7
718.9
719.8
239.4
240.2
528.3
527.3
279.5
591.5
720.8
1254.2
284.2
1252.7
753.8
389.7
895.8
1332.0
1390.8
1902.9
1947.8
840.9
985.9
1518.6
1761.2
1953.2
200
400
600
800
1000
1200
1400
1600
1800
2000
m/z

Probe MA-β-gal-cyclobutyl

^{1}H-NMR

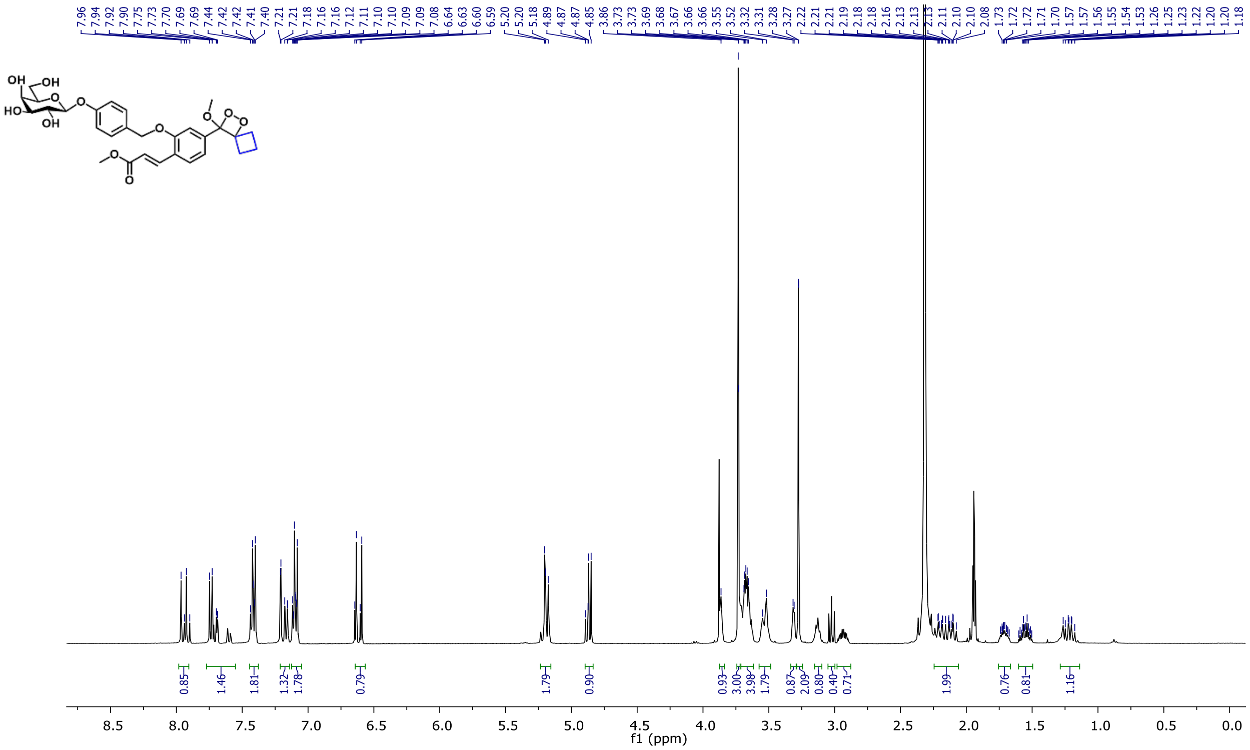

^{13}C-NMR

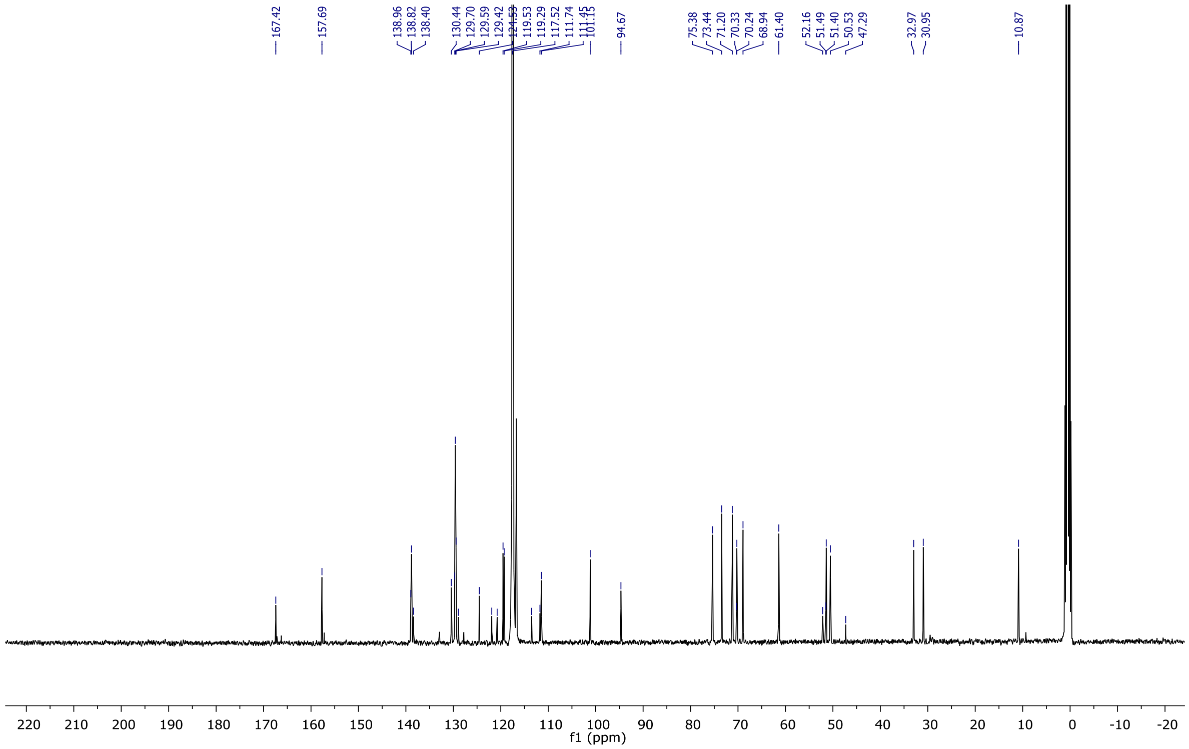

MS

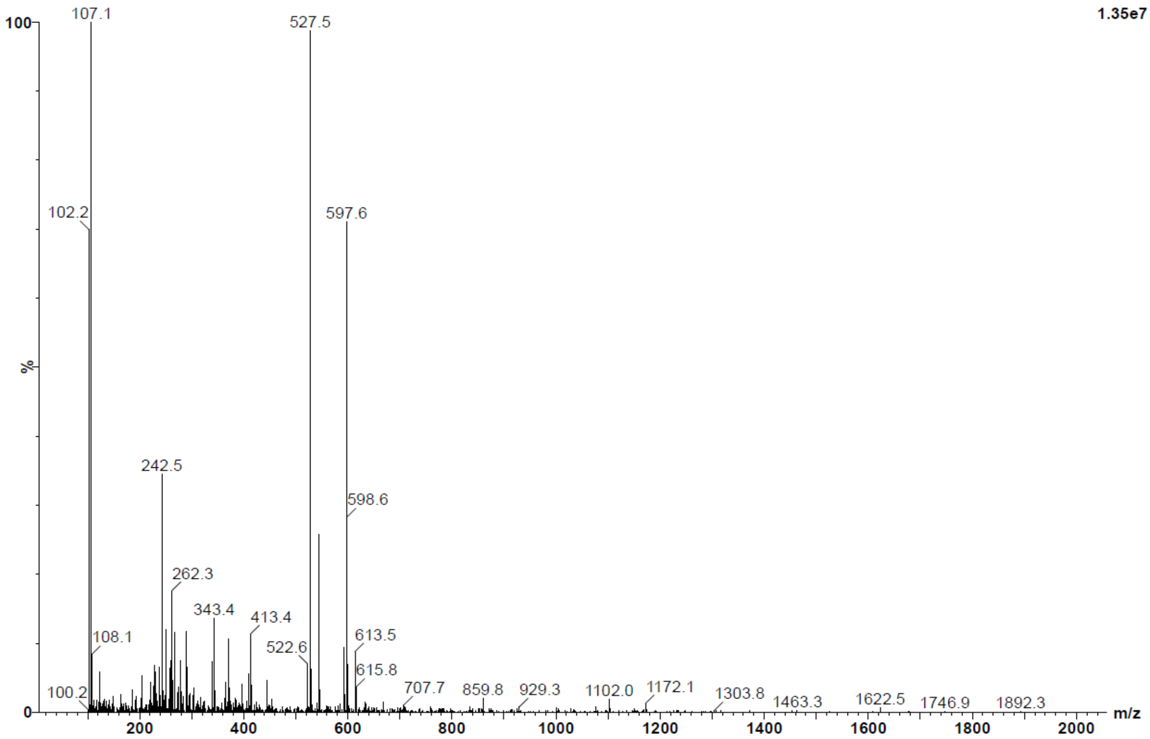
107.1
527.5
1.35e7
100
102.2
597.6
%
242.5
598.6
262.3
343.4
413.4
108.1
522.6
613.5
615.8
100.2
707.7
859.8
929.3
1102.0
1172.1
1303.8
1463.3
1622.5
1746.9
1892.3
0
200
400
600
800
1000
1200
1400
1600
1800
2000
m/z

References

(1) Green, O.; Eilon, T.; Hananya, N.; Gutkin, S.; Bauer, C. R.; Shabat, D. Opening a Gateway for Chemiluminescence Cell Imaging: Distinctive Methodology for Design of Bright Chemiluminescent Dioxetane Probes. *ACS Cent. Sci.* **2017**, *3*, 349-358.

(2) Tannous, R.; Shelef, O.; Gutkin, S.; David, M.; Leirikh, T.; Ge, L.; Jaber, Q.; Zhou, Q.; Ma, P.; Fridman, M. et al; Spirostrain-Accelerated Chemiexcitation of Dioxetanes Yields Unprecedented Detection Sensitivity in Chemiluminescence Bioassays. *ACS Cent. Sci.* **2024**, *10* (1), 28– 42.

(3) *Gaussian 16, Revision C.01, Frisch, M. J.; Trucks, G. W.; Schlegel, H. B.; Scuseria, G. E.; Robb, M. A.; Cheeseman, J. R.; Scalmani, G.; Barone, V.; Petersson, G. A.; Nakatsuji, H.; Li, X.; Caricato, M.; Marenich, A. V.; Bloino, J.; Janesko, B. G.; Gomperts, R.; Mennucci, B.; Hratchian, H. P.; Ortiz, J. V.; Izmaylov, A. F.; Sonnenberg, J. L.; Williams-Young, D.; Ding, F.; Lipparini, F.; Egidi, F.; Goings, J.; Peng, B.; Petrone, A.; Henderson, T.; Ranasinghe, D.; Zakrzewski, V. G.; Gao, J.; Rega, N.; Zheng, G.; Liang, W.; Hada, M.; Ehara, M.; Toyota, K.; Fukuda, R.; Hasegawa, J.; Ishida, M.; Nakajima, T.; Honda, Y.; Kitao, O.; Nakai, H.; Vreven, T.; Throssell, K.; Montgomery, J. A., Jr.; Peralta, J. E.; Ogliaro, F.; Bearpark, M. J.; Heyd, J. J.; Brothers, E. N.; Kudin, K. N.; Staroverov, V. N.; Keith, T. A.; Kobayashi, R.; Normand, J.; Raghavachari, K.; Rendell, A. P.; Burant, J. C.; Iyengar, S. S.; Tomasi, J.; Cossi, M.; Millam, J. M.; Klene, M.; Adamo, C.; Cammi, R.; Ochterski, J. W.; Martin, R. L.; Morokuma, K.; Farkas, O.; Foresman, J. B.; Fox, D. J. Gaussian, Inc., Wallingford CT,* 2016. (accessed 2023-07-07).

(4) Chai, J. D.; Head-Gordon, M. Long-range corrected hybrid density functionals with damped atom-atom dispersion corrections. *Phys. Chem. Chem. Phys.* **2008**, *10*, 6615-6620.

(5) Clark, T.; Chandrasekhar, J.; Sp itznagel, G. W.; Schleyer, P. V. Efficient Diffuse Function-Augmented Basis Sets for Anion Calculations. Iii. The 3-21+G Basis Set for First-Row Elements, Li-F. *J. Comput. Chem.* **1983**, *4*, 294-301.

(6) Krishnan, R.; Binkley, J. S.; Seeger, R.; Pople, J. A. Self-Consistent Molecular-Orbital Methods .20. Basis Set for Correlated Wave-Functions. *J. Chem. Phys.* **1980**, *72*, 650-654.

(7) Marenich, A. V.; Cramer, C. J.; Truhlar, D. G. Universal Solvation Model Based on Solute Electron Density and on a Continuum Model of the Solvent Defined by the Bulk Dielectric Constant and Atomic Surface Tensions. *J. Phys. Chem. B* **2009**, *113*, 6378-6396.

(8) Gonzalez, C.; Schlegel, H. B., Reaction path following in mass-weighted internal coordinates. *J. Phys. Chem*. **1990**, *94* (14), 5523-5527.

(9) Yanai, T.; Tew, D. P.; Handy, N. C. A new hybrid exchange-correlation functional using the Coulomb-attenuating method (CAM-B3LYP). *Chem. Phys. Lett.* **2004**, *393*, 51-57.

(10) Grimme, S.; Antony, J.; Ehrlich, S.; Krieg, H. A consistent and accurate ab initio parametrization of density functional dispersion correction (DFT-D) for the 94 elements H-Pu. *J. Chem. Phys.* **2010**, *132*, 154104.

(11) Grimme, S.; Ehrlich, S.; Goerigk, L. Effect of the Damping Function in Dispersion Corrected Density Functional Theory. *J. Comput. Chem.* **2011**, *32*, 1456-1465.

(12) Grimme, S. Supramolecular Binding Thermodynamics by Dispersion-Corrected Density Functional Theory. *Chem. Eur. J.* **2012**, *18*, 9955-9964.

(13) Luchini, G.; Alegre-Requena, J. V.; Funes-Ardoiz, I.; Paton, R. S. GoodVibes: automated thermochemistry for heterogeneous computational chemistry data. *F1000research* **2020**, *9*, 291.
